# Small-Molecule PROTACs for Cancer Immunotherapy

**DOI:** 10.3390/molecules27175439

**Published:** 2022-08-25

**Authors:** Zefan Liu, Yajun Zhang, Yucheng Xiang, Xin Kang

**Affiliations:** West China (Airport) Hospital, Sichuan University, Chengdu 610047, China

**Keywords:** targeted protein degradation, proteolysis targeting chimera, tumor immunotherapy, small molecule inhibitors, tumor microenvironment

## Abstract

Unsatisfactory physicochemical properties of macromolecular drugs seriously hinder their application in tumor immunotherapy. However, these problems can be effectively solved by small-molecule compounds. In the promising field of small-molecule drug development, proteolysis targeting chimera (PROTAC) offers a novel mode of action in the interactions between small molecules and therapeutic targets (mainly proteins). This revolutionary technology has shown considerable impact on several proteins related to tumor survival but is rarely exploited in proteins associated with immuno-oncology up until now. This review attempts to comprehensively summarize the well-studied and less-developed immunological targets available for PROTAC technology, as well as some targets to be explored, aiming to provide more options and opportunities for the development of small-molecule-based tumor immunotherapy. In addition, some novel directions that can magnify and broaden the protein degradation efficiency are mentioned to improve PROTAC design in the future.

## 1. Introduction

The recent decades witnessed the bloom of tumor immunotherapy [1,2,3]. The central aim of immunotherapy is to harness autologous immune responses for tumor elimination [1,4,5,6,7,8]. Distinct from conventional approaches such as surgery, chemotherapy, and radiotherapy, the modulation of the immune system can lead to abscopal and long-lasting therapeutic consequences, therefore preventing tumor recurrence and metastasis [9,10,11]. Hitherto, most clinically approved immune-intervening agents are macromolecules, such as blockade antibodies, engineered immune cells, oncolytic viruses, cytokines/chemokines, and vaccines [2,10,12]. Though benefiting from treatments, there are some problems with these drugs [9,13]. Firstly, because of the low oral bioavailability, macromolecular drugs are often intravascularly administrated, leading to poor patient compliance [2,9]. Secondly, macromolecular drugs exhibit a low clearance rate and a long half-life, resulting in uncontrollable organ distribution and pharmacokinetics [13,14]. Thirdly, macromolecular drugs have the poor capability in tissue penetration and transmembrane transport, which seriously hinders their effects in dense solid tumors [14]. Lastly, macromolecular drugs bear a risk of eliciting immune-related adverse events (irAEs) [13].

As the dominant proportion in classical antitumor therapies, small-molecule drugs offer an ideal approach to addressing the above problems [15]. Several reviews have recapitulated the advantages of small molecules over macromolecules in immuno-oncology, which can be attributed to the following aspects: (1) small molecules can be orally administrated [14,15]; (2) small molecules are eliminated more rapidly than macromolecules, which allows for precise prescription and flexible treatment regime [14]; (3) small molecules had better membrane permeability than macromolecular drugs, so they can act on intracellular targets to orchestrate intricate signal pathways [9,15]; they are also more capable of overcoming extracellular barriers, favoring drug accumulation in dense tumor tissues [15]; (4) small molecules take lower cost in manufacture, storage, transport, and medication than macromolecular drugs [9]. 

Proteolysis targeting chimera (PROTAC) is a leading field in the discovery of small-molecule drugs [16,17]. A ubiquitin-proteasome system (UPS) is the natural machinery for the degradation of cellular proteins [18]. Ubiquitin is transferred to the surface lysine of substrate proteins by a cascade of three enzymes: ubiquitin-activating enzymes (E1), ubiquitin-conjugating enzymes (E2) and ubiquitin-protein ligases (E3), wherein the E3 ligase play pivotal roles [16,18,19]. Ubiquitin also contains lysine residues that can be consecutively ubiquitinated to polyubiquitin chains. Subsequently, the polyubiquitin-tagged proteins are recognized by proteasomes for degradation. The major concept of PROTAC is to hijack UPS to specifically degrade pathogenic or abnormally overexpressed proteins [18]. Since firstly proposed in 2001 by Craig M. Crews, this technology has revolutionized the mechanisms of drug-protein interactions in the past two decades [16,18,19,20]. Most compounds competitively bind with the catalytic site, inhibit kinase phosphorylation, or allosterically decrease the activity of functional proteins [21,22,23,24]. 

**Figure 1:**
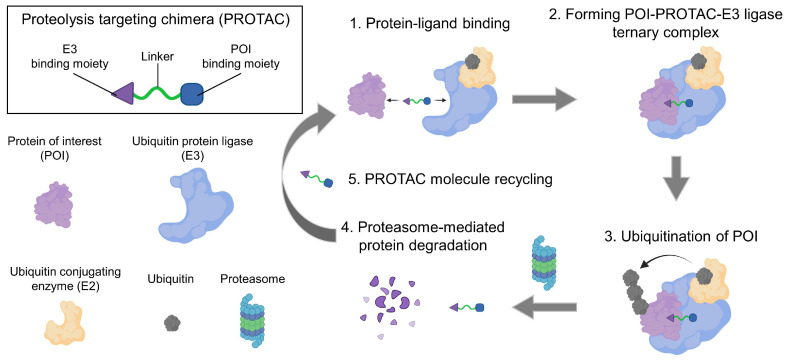
Schematic illustration of the mechanism of PROTAC. Firstly, the PROTAC molecule binds with POI and E3 ligase using the functional groups in its two ends. Then, the PROTAC molecule joins these two proteins together, forming a complex consisting of POI, PROTAC, and E3 ligase. The proximity of POI to the E3 ligase enables the ubiquitination of POI. Subsequently, the ubiquitin-tagged protein is recognized and degraded by the proteasome, and the PROTAC molecule can be reused to connect the next POI and E3 ligase.

On the basis of these interactions, PROTAC molecules bring a protein of interest (POI) into degradation [16]. PROTACs are heterobifunctional compounds that consist of three moieties [19,25]: (1) a warhead for POI to be degraded, (2) a ligand to engage the E3 ubiquitin ligase, and (3) a flexible linker joining them. Two ends of the PROTAC molecules bind with their targets, respectively, forming a POI-PROTAC-E3 ligase ternary complex [26]. After that, the POI is ubiquitinated and degraded by the proteasome (Figure 1) [16,18].

Nowadays, more than 600 E3 ligases have been identified. Small molecule ligands for some of these E3 ligases, such as mouse double minute 2 (MDM2), cell inhibitor of apoptosis (cIAP), von Hippel–Lindau protein (pVHL), Cereblon (CRBN), Kelch-like ECH-associated protein 1 (KEAP1), aryl hydrocarbon receptor (AhR), DDB1-CUL4 associated factor 11/15/16 (DDAF11/15/16), ring finger protein 4/144 (RFN4/144) and fem-1 homolog B (FEM1B) have also been discovered [27]. Among these various E3 ligase ligands, CRBN and VHL ligands are the best options in PROTAC design because of several aspects [28,29]. Firstly, CRBN and VHL are widely and abundantly expressed in multiple tumor cells, which guarantees the degradation efficiencies of CRBN- and VHL-based PROTACs. Secondly, CRBN and VHL ligands are easy to synthesize, and their molecule weights are relatively low, exhibiting good drug-like properties [30]. Finally, the safety and biocompatibility of these two kinds of ligands have been extensively evaluated in vivo. 

PROTAC technology has many advantages over conventional small-molecule inhibitors [13,25]. Undruggable proteins refer to proteins without well-defined binding pockets, which occupy 80% of disease-associated proteins [19]. These proteins, including non-enzymatic proteins, scaffolding proteins, and transcription factors, are not reactive to small-molecule inhibitors but can be degraded by PROTACs [19,31]. That is because PROTAC molecules can bind to anywhere of the POI with moderate affinity for protein degradation, but classical small-molecule inhibitors need to interact with the binding pocket with high affinity for function [16,32]. Moreover, tumor cells easily acquire resistance via protein mutation after long-term drug exposure, which leads to treatment failure. Studies showed that drug-resisting tumor cells are resistant to small-molecule inhibitors but vulnerable to PROTAC compounds, denoting the capability of PROTAC to obviate drug resistance [26]. To suppress protein activity, small-molecule inhibitors have to permanently couple with their targets. Eliciting a stoichiometric drug response [25]. In contrast, noncovalently tethered PROTAC molecules can be recovered and joined into the next circulation soon after protein degradation by the proteasome [19,25]. Collectively, as the next generation of small-molecule therapies, PROTAC-based drugs are expected to thoroughly replace macromolecular therapies in tumor immunotherapy [25,32,33,34].

Despite the attractive prospect of applying PROTAC to immuno-oncology, few studies have focused on this issue, which might be attributed to the immature concepts and approaches. There are some existing reviews that refer to targeted protein degradation (TPD) strategies for tumor immunotherapy, but they merely focus on molecule designs and their applications in programmed cell death 1 (PD-1)/programmed death ligand 1 (PD-L1) immune checkpoint [33,34]. The influence of PROTAC molecules in multiple events within the tumor microenvironment (TME), such as signal crosstalk, antigen presentation, immune cell invasion and tumor immunogenicity, are not mentioned in these review articles [4,33,34,35,36]. Thus, it is required to summarize the current advances and discover more targets in PROTAC-mediated tumor immunotherapy. 

To this end, this review gathered the application of PROTACs for immune-related targets in recent three years (Table 1), including the PD-1/PD-L1 checkpoint and its regulatory pathways (SHP2 and BET), vital processes in tumorigenesis such as metabolism (IDO1), epigenetic modification (HDAC), and apoptosis (Bcl-2 family), as well as immuno-modulating signals (STAT3 and MAPK), and also some targets which have not or rarely treated by PROTAC molecules (CD47, Foxp3, COX-1/2, NAMPT, and TGF-β1). It should be noted that most of these targets and PROTACs were summarized by Rao et al. in recent reviews [28,37]. Thus, this review will specifically focus on the immunological consequences after the degradation/inhibition of these targets, aiming to bias the development of small-molecule PROTACs for immunomodulation rather than simple tumor killing. In this article, we hope to provide comprehensive knowledge about the principle of PROTAC-mediated immune intervention and boost the discovery of versatile PROTAC molecules for tumor immunotherapy.

## 2. Small-Molecule PROTACs Targeting PD-1/PD-L1 Checkpoint Signal Axis

### 2.1. Small-Molecule PROTACs Targeting PD-L1 Protein

**Figure 2:**
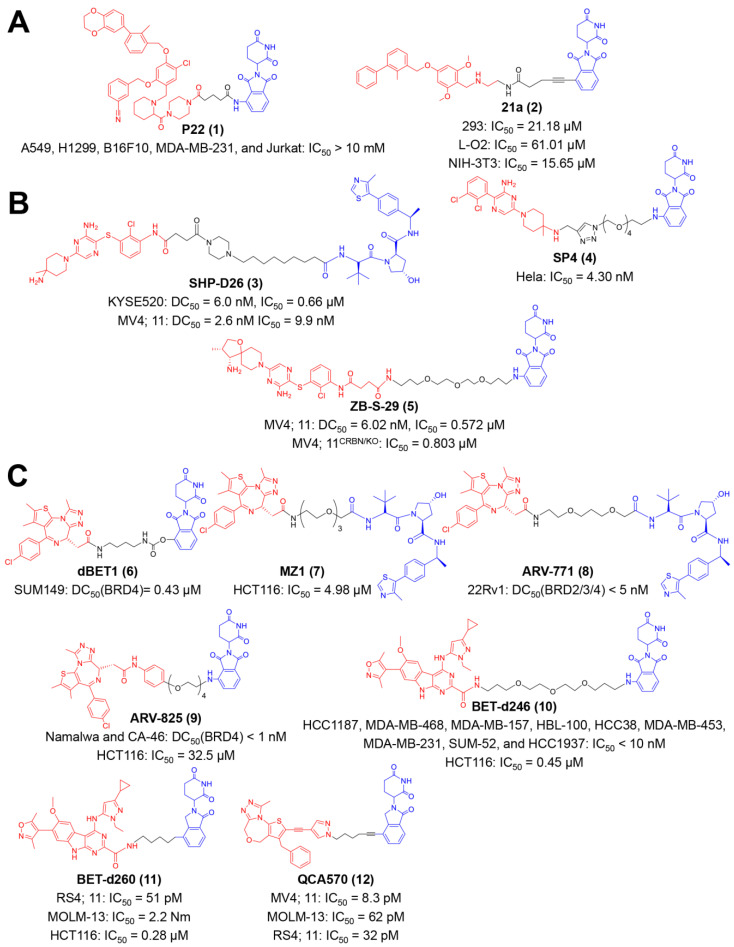
The chemical structures of representative PROTACs targeting the PD-1/PD-L1 checkpoint signal axis. (**A**) The chemical structures of representative PROTACs targeting PD-L1 protein. (**B**) The chemical structures of representative PROTACs targeting SHP-2. (**C**) The chemical structures of representative PROTACs targeting BET. DC_50_—half-maximal degradation concentration; IC_50_—half-maximal inhibition concentration.

Cancer cells express PD-L1 on their surface to evade cytotoxic T lymphocytes (CTLs)-mediated tumor killing [84,85,86]. Cumulative evidence has shown the superiority of targeted degradation on long-term PD-L1 dysfunction over conventional antibody-based blockade [34,87,88,89,90]. The first PROTAC-like small molecule for PD-L1 degradation was reported by Chen and co-workers (Figure 2A) [38]. Resorcinol diphenyl ethers were utilized as the PD-L1 targeting group, and pomalidomide was utilized as the ligand to the E3 ubiquitin ligase [38]. They found compound P22 exhibited distinguished inhibitory activity on PD-1/PD-L1 interaction among a series of synthesized compounds, with a half-maximal inhibition concentration (IC_50_) of 39.2 nM [38]. P22 did not directly inhibit the growth of tumor cells (IC_50_ values > 10 mM for A549, H1299, B16F10, MDA-MB-231, and Jurkat cell lines), but the treatment of P22 could restore the immune response of CD3^+^ T-cells to Hep3B/OS-8/hPD-L1 tumor cells [38]. Other PROTAC molecules were reported by Yang et al. (Figure 2A) [39]. The diaryl ether molecule BMS-37, with high PD-L1 inhibition activity, was chosen as the targeting ligand, and pomalidomide was also leveraged as yjr E3 ubiquitin ligase-oriented group [39]. A library of PROTAC compounds was synthesized with different linkers, and their PD-L1 degrading ability was assayed by Western blot. Western blot assays showed that compound **21a** potently decreased PD-L1 expression on MC38 cells in a proteasome-dependent manner [39]. 21a had a relative less cytotoxicity on normal cells, such as 293 (IC_50_ = 21.18 μM), L-O2 (IC_50_ = 61.01 μM), and NIH-3T3 (IC_50_ = 15.65 μM), indicating a well safety [39]. In vivo studies revealed that 21a had anti-tumor activity on MC38 xenografts, which was beneficial from the PD-L1 downregulation and the spurring of CD8^+^ T-cells [39]. 

### 2.2. Small-Molecule PROTACs Targeting SHP2

Src homology-2 domain-containing protein tyrosine phosphatase (SHP2) is a multifunctional tumorigenic protein with a central role in the survival, proliferation, metastasis, and drug resistance of tumor cells [91,92,93,94]. Recent studies have uncovered the effect of SHP2 on T-cell exhaustion in a tumor microenvironment [95,96]. After binding to PD-L1, PD-1 recruits SHP2 via the phosphorylation of its tyrosine-containing motifs, subsequently inducing a cascade of downstream pathways for T-cell suppression, such as abrogation of the T-cell receptor (TCR) and CD28, deprivation of cytokine production and impairment of T-cell proliferation [91,93,95,97]. Hence, targeting SHP2 might significantly potentiate PD-1/PD-L1 blockade therapy. Several small-molecule SHP2 inhibitors have shown immuno-boosting activities [92,98]. Hao et al. reported the synergistic effect of the first clinically-tested allosteric SHP2 inhibitor—TNO155—with the PD-L1 antibody in preclinical studies [99]. Methylene blue, an FDA-approved drug for methemoglobinemia treatment, was found to inhibit PD-1 and restore the activities of CTLs, wherein the mechanism was associated with blocking SHP2 recruitment by PD-1 [100]. Another allosteric SHP2 inhibitor, SHP099, was also discovered to augment immune response against the CT26 tumor model in vivo with the dual function of normalization and enhancement [101]. The therapeutic effects of SHP2 inhibition were further investigated beyond the PD-1/PD-L1 axis. Research from Smith et al. suggested that the allosteric inhibitor RMC-4550 not only elicits a greater antitumor immunity than checkpoint blockade but also depletes M2 macrophages and increases M1 phenotype, reversing the immunosuppressive tumor microenvironment [102]. Moreover, studies by Kulkarni et al. showed that SHP2 inhibition could increase the phagocytosis potential of macrophages [101]. 

As a novel protein degradation approach, PROTAC offers a more effective approach to orchestrating SHP2-mediated immunity [103]. Wang et al. discovered the first PROTAC degrader of SHP2 protein (Figure 2B) [40]. The SHP2-binding group was inspired by the above-mentioned inhibitor, SHP099, and the VHL ligand was employed for cullin 2 E3 ligase complex recruiting [40]. The compound SHP-D26 potently degrades the SHP2 protein in esophageal cancer KYSE520 and acute myeloid leukemia (AML) MV4; the 11-cell line, with a half-maximal degradation concentration (DC_50_) of 6.0 and 2.6 nM, respectively. Furthermore, this compound inhibited tumor cell proliferation for over 30 folds in comparison with SHP099, with an IC_50_ value of 0.66 μM in KYSE520 and 9.9 nM in MV4; 11 cells [40]. Other PROTAC compounds based on SHP099 were reported by Li et al. (Figure 2B) [41]. They coupled pomalidomide to SHP099 via azide-PEG-amine linkers and obtained four compounds with various PEG lengths [41]. Among these compounds, SP4 exhibited a 100-fold elevated cytostatic effect than the SHP099 on Hela cells (IC_50_ = 4.30 nM) and effectively triggered SHP2 degradation and cell apoptosis [41].

Zhou et al. exemplified another CRBN-based PROTAC degrader of SHP2 (Figure 2B) [42]. An analogue of TNO155, a powerful SHP2 inhibitor, was employed to recruit SHP2 protein, and thalidomide was chosen as the CRBN-hijacking binder [42]. A variety of compounds with different linker lengths, compositions and tethering sites were synthesized [42]. They identified ZB-S-29 as the ideal SHP2 degrader with a DC_50_ value of 6.02 nM, as well as a suitable cytotoxic agent (IC_50_ = 0.572 μM in MV4; 11 cells).

### 2.3. Small-Molecule PROTACs Targeting BET

The bromodomain and extra-terminal domain (BET) protein family plays a vital role in epigenetic and transcriptional regulation [104,105,106]. Its immuno-regulating function is mainly associated with PD-L1 expression, which is mediated by bromodomain-containing protein 4 (BRD4) [104,105,107]. Small-molecule BET inhibitors (BETi) are demonstrated to noticeably reduce PD-L1 in mRNA and protein levels in several cancers, such as melanoma, triple-negative breast cancer (TNBC), liver cancer, and lung cancer [108,109]. In addition, BETi (e.g., JQ1) works beyond PD-L1 downregulation. Effects such as increasing the immunogenicity of tumor cells, stimulating CD8^+^ T-cells and anti-inflammation are also found after BETi treatments, which strongly potentiates antitumor immunity [110,111]. 

Small-molecule PROTACs for BET protein have been developed extensively for many years, and several compounds have manifested considerable therapeutic outcomes (Figure 2C). In 2015, Bradner et al. appended JQ1 to the phthalimide moiety, obtaining dBET1 as a CRBN-based PROTAC (Figure 2C) [43]. dBET1 specifically induced BRD4 degradation (DC_50_ = 0.43 μM in SUM149 cells) both in vitro and in vivo, and retarded leukemia in mice models [43]. This year also witnessed the appearance of a VHL-based substitute of dBET1, reported by Ciulli et al., termed MZ1 (Figure 2C) [44]. The Western blot assay and gene expression profiles validated the reversible, long-lasting and selective suppression of BRD4 elicited by MZ1 [45]. Furthermore, its capability on PD-L1 downregulation was verified in the head and neck squamous cell carcinoma (HNSCC) cell line [44].

An analogue of MZ1 with a shorted linker, ARV-771, was developed by Coleman et al. (Figure 2C) [46]. ARV-771 effectively degraded BRD2/3/4 in the 22Rv1 cell line with a DC_50_ value < 5 nM [43]. Importantly, they demonstrated the efficacy of a small-molecule BET degrader to the solid tumor (castration-resistant prostate cancer), xenograft model for the first time [46]. This group also explored the feasibility of another BET-binding moiety in PROTAC design at early times (Figure 2C) [47,48]. The resultant compound ARV-825 uses OTX015 as the warhead towards BRD4 and pomalidomide as the CRBN-interacting group [47,48]. ARV-825 responded well to hematologic malignancies, such as Burkitt’s lymphoma (BL) and multiple myeloma (MM). In terms of immunotherapy, ARV-825 could activate natural killer (NK) cells and improve the sensitivity of MM cells to NK cell-mediated cytolysis [47]. Wang et al. identified azacarbazoles (HJB97) as a novel class of BETi, and accordingly discovered a library of thalidomide/lenalidomide-based BET degraders (Figure 2C) [49,51]. Among these compounds, BETd-246 and BETd-260 performed excellently in protein degradation and tumor regression on multiple mice and patient-derived xenograft (PDX) models [49,50,51]. Mechanistically, these two compounds trigger immunogenic cell death (ICD) via BET degradation, which facilitates PD-1 blockade immunotherapy [49,50,51]. In 2018, this group discovered 1,4-Oxazepines as another new class of BETi via structure-guided design and subsequently proposed a series of CRBN-based PROTACs (Figure 2C) [52]. More than potent BET degradation, QCA570 from these compounds achieves cell growth inhibition with IC_50_ values of 8.3, 62, and 32 pM in MV4; 11, MOLM-13, and RS4; 11 cells, respectively, and also lastingly suppresses leukemia in xenograft models [52].

## 3. Small-Molecule PROTACs for Vital Regulatory Proteins

### 3.1. Small Molecule PROTACs for IDO1

**Figure 3:**
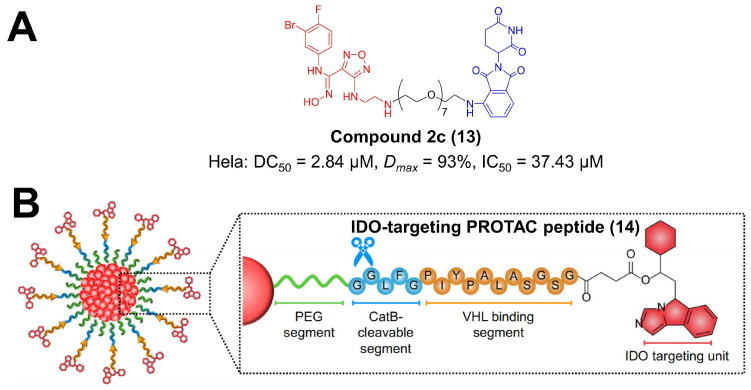
The chemical structures of representative PROTACs targeting IDO1. (**A**) The chemical structure of the first PROTAC targeting IDO1. (**B**) The chemical structure of IDO-targeting PROTAC peptide. DC_50_—half-maximal degradation concentration; *Dmax*—maximum degradation; IC_50_—half-maximal inhibition concentration.

Indoleamine 2, 3-dioxygenase 1 (IDO1) is a rate-limiting enzyme in the kynurenine pathway and is highly overexpressed in malignant cells, leading to tryptophan exhaustion and kynurenine accumulation in the tumor milieu [112]. The unbalanced tryptophan metabolism reprograms T cells towards an immunosuppressive regulatory phenotype, assisting tumor immune escape [112,113]. A great number of IDO1 inhibitors with diverse structures have been developed for cancer treatment, and some of them are being proceeded into clinical trials [113,114,115]. In order to improve specificity and minimize the side effects of inhibitors, researchers attempted to apply PROTAC for IDO1 blockade [114,116,117,118,119]. In 2020, Xie et al. reported the first PROTAC degrader for IDO1 protein (Figure 3A) [53]. They choose epacadostat as the preferable IDO1 ligand, pomalidomide/lenalidomide as the E3 ligase ligand, and amphiphilic polyethylene glycol (PEG) with various repeat units as the linker [116]. Among these designed PROTACs, compound **2c** exerted a satisfactory and durable effect on IDO1 degradation via UPS with a DC_50_ of 2.84 μM in Hela cells [53]. Notably, compound **2c** moderately reinforced the lethal activity of chimeric antigen receptor-modified T (CAR-T) cells in tumor cells [53]. PROTAC strategies can be incorporated with nanotechnology for IDO1 degradation [54]. Pu et al. designed an IDO-targeting PROTAC peptide (IPP), in which IDO inhibitor NLG919 was connected with a VHL-binding sequence via a succinic linker (Figure 3B) [54]. IPP was further conjugated to semiconductor polymer core via tumor-cleavable peptide, forming a nano-PROTAC [54]. This nanoplatform significantly activated effector T-cells via persistent IDO1 degradation, dually inhibiting tumor growth and metastasis [54]. It is suggested that epidermal growth factor receptor (EGFR) upregulated IDO1 and PD-L1 in cancerous diseases [119]. Small-molecule PROTACs for EGFR are demonstrated to decline IDO1 as well as PD-L1 in non-small cell lung cancer (NSCLC) cell lines and tumor tissues [119]. This research indicates that the IDO1 checkpoint can be manipulated by PROTAC technologies.

### 3.2. Small-Molecule PROTACs Targeting HDAC

Histone deacetylase (HDAC) are key epigenetic regulators which transcriptionally silences the chromatin by removing the acetyl group from lysine residues on the histone tail [120]. Inhibitors of HDAC (HDACi) could restore and increase the transcriptional activity of tumor-suppressive genes to exert miscellaneous anticancer effects [120,121]. Since immune-associated genes (such as immune recognition and antigen presentation) are also epigenetically modulated by HDAC, versatile immune intervening effects of HDACi have been identified [121]. On the one hand, HDACi highly elevated the response of tumor cells to immune cells [122]. Domatinostat, AR42 and sodium valproate upregulated the expression of class I major histocompatibility complex molecules (MHC I) on tumor cells, facilitating their phagocytosis by dendritic cells (DCs) [123]. Romidepsin induced the secretion of various chemokines from tumor cells, such as CCL5, CXCL9 and CXCL 10, attracting T lymphocytes into the tumor [124]. Vorinostat and entinostat increased the expression of multiple death receptors on tumor cells, making them more vulnerable to the killing effects mediated by NK cells and antibodies [125]. In addition, several HDACi such as MPT0G612, AR42 and sodium valproate downregulated PD-L1 on tumor cells, exerting a synergistic effect with checkpoint blockade therapy [126]. On the other hand, HDACi significantly reversed immunosuppressive TME and augmented the antitumor activity of tumor-infiltrated immune cells [123]. Entinostat decreased the intra-tumoral infiltration of myeloid-derived suppressor cells (MDSCs) in breast and pancreatic tumor models [127]. Mocetinostat was reported to reduce both regulatory T-cells (Tregs) and MDSCs in tumors [128]. In addition to the aforementioned functions, another HDACi, CG-745, suppressed M2 macrophage polarization and promoted the proliferation of T lymphocytes [129]. More than sensitizing tumor cells, vorinostat directly elevated the active stage of NK cells against malignant diseases [125]. Overall, HDAC inhibitors are very promising in interrupting the crosstalk between tumor cells and immune cells in TME. 

**Figure 4:**
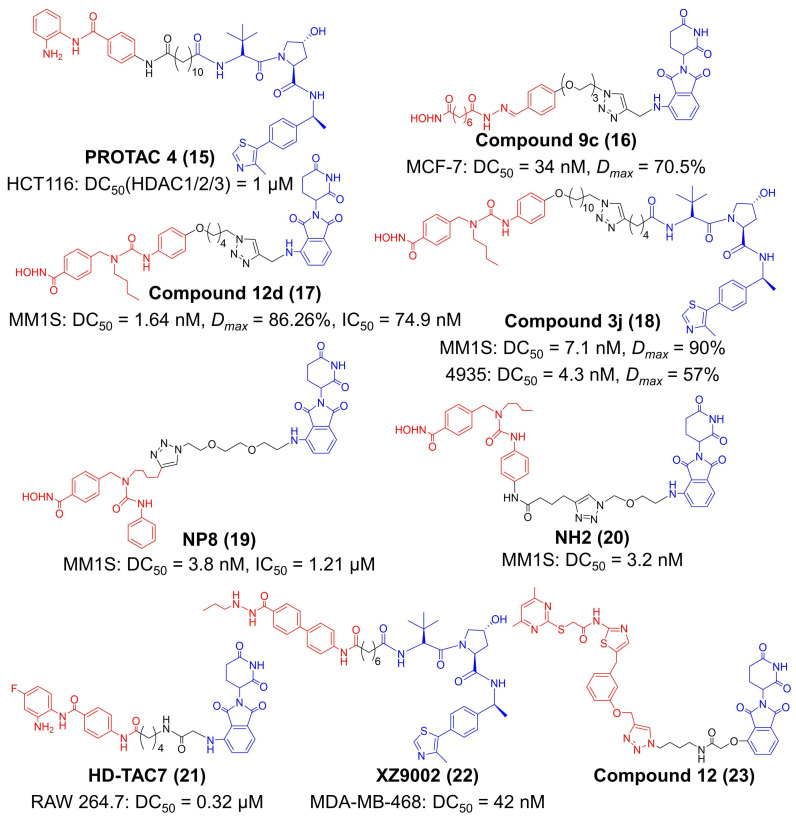
The chemical structures of representative PROTACs targeting HDAC. DC_50_—half-maximal degradation concentration; *Dmax*—maximum degradation; IC_50_—half-maximal inhibition concentration.

Up to now, eighteen members have been identified in the HDAC family, and they are classified according to their homology to yeast proteins [130]. Eleven members of the HDAC family have a Zn^2+^ in their catalytic sites, and they are divided into three groups: class I (including HDAC 1, 2, 3, and 8), class IIa (including HDAC 4, 5, 7, and 9), class IIb (including HDAC 6 and 10), and class IV (HDAC 11) [120]. Another seven members of the HDAC family are NAD^+^ dependent enzymes without Zn^2+^ in catalytic sites—they belong to class III HDAC, which is also termed as silent information regulator 2-related enzymes (sirtuins) [130]. Since the non-selective HDACi often cause undesirable toxicity, specifically degrading one or more certain HDAC members by PROTAC may improve the antitumor activities and immune-modulating capacities of HDACi [130]. Hodgkinson et al. designed a PROTAC for class I HDAC (Figure 4) [55]. This compound (PROTAC 4) utilized a benzamide-based structure as the ligand to HDAC 1, 2 and 3, and a VHL ligand was connected with an alkyl linker [55]. PROTAC 4 had an IC_50_ value of 16.8 μM against the LSD1-CoREST-HDAC1 complex and degraded 50% of HDAC1, HDAC2 and HDAC3 at 1 μM in HCT116 cells [127]. Treatment of PROTAC 4 successfully elevated the acetylation level of histone and compromised cell viability [55]. 

Concurrent degradation of several HDACs still has a risk of unsatisfactory effects [56]. Hence, targeted degradation of the key pathogenic HDAC subtypes becomes a preferable option [56,131,132]. Unlike conventional HDAC proteins that localize in the nucleus, HDAC6 mainly distributes in the cytoplasm and interacts with multiple cytosolic nonhistone proteins, including α-tubulin, and heat shock protein 90 (HSP90) and cortactin [133]. It is well-documented that HDAC6 upregulated PD-L1 on melanoma cells [133,134]. The first small-molecule PROTAC for HDAC6 was reported by Tang et al. in 2018 (Figure 4) [56]. They conjugated pan-HDAC inhibitor vorinostat and compound **2c** to thalidomide analogues, finding that this approach turns the compounds into selective HDAC6 degraders (compound **9c**) [56]. Compound **9c** achieved a DC_50_ of 34 nM and a maximum degradation (*Dmax*) of 70.5% in MCF-7 cells [56]. To further improve the degradation efficiency, they developed another two PROTAC compounds based on Nexturastat A (Nex A), a selective HDAC6 inhibitor, in 2019 (compound **12d**) and 2020 (compound **3j**) (Figure 4) [57,58,135]. These two compounds employed CRBN and VHL E3 ubiquitin ligases for HDAC6 degradation, respectively [108,109]. Compound **12d** had a IC_50_ of 8.7 nM against HDAC6 [57]. The DC_50_ and *Dmax* of compound **12d** were 1.64 nM and 86.26% against MM1S cells, and compound **12d** also had an antiproliferation effect in MM1S cells with a IC_50_ of 74.9 nM [57]. The DC_50_ and *Dmax* of compound **3j** was 7.1 nM and 90% in human MM1S cells, while 4.3 nM and 57% in murine 4935 cells [58]. Inspired by the Y-shaped conformation of Nex A when binding to HDAC6, Rao et al. managed to conjugate pomalidomide at different sites of Nex A, obtaining two potent HDAC6 degraders, NP8 and NH2 (Figure 4) [59,60]. The DC_50_ value of NP8 and NH2 in MM1S cells was 3.8 and 3.2 nM, respectively [59,60]. Their work highly expanded the versatility and plasticity of HDAC6 inhibitor-based PROTACs. 

In addition to HDAC6, HDAC3 is also involved in the immune response by affecting the NF-κB signal [61,62]. Accordingly, Dekker et al. tethered the CRBN ligand pomalidomide to clinically-trailed HDACi CI994 with varied linkers to synthesize HDAC degraders (Figure 4) [61]. Among these products, HD-TAC7 had higher activity in HDAC3 (IC_50_ = 1.1 μM) than HDAC1 (IC_50_ = 3.6 nM) and HDAC2 (IC_50_ = 4.2 μM) and exhibited excellent degradation of HDAC3 (DC_50_ = 0.32 μM) in RAW264.7 macrophages [61]. Liao et al. identified SR-3558 as a potent HDAC3 inhibitor and synthesized a library of PROTAC molecules based on SR-33558 (Figure 4) [62]. One of the VHL-recruiting degraders, XZ9002, exhibited the most effective HDAC3 degradation in MDA-MB-468 cells (DC_50_ = 42 nM) [62]. Sirt2, one of class Ⅲ HDACs, was proved to participate in autophagy, inflammation and immune response [63]. To develop PROTAC molecules for Sirt2, Jung et al. attached thalidomide to sirtuin rearranging ligands (SirReals) (Figure 4) [63]. The SirReal-Based PROTAC compound **12** induced isotype-selective inhibition with a IC_50_ value of 0.25 μM in Sirt2 but >100 uM in Sirt1 and Sirt3.

### 3.3. Small Molecular PROTACs Targeting the Bcl-2 Family

The B-cell lymphoma-2 (Bcl-2) protein family are pivotal regulators in apoptosis and is highly associated with tumor progress [136,137]. Structurally, proteins in the Bcl-2 family share one to four conserved regions, which are called Bcl-2 homology (BH) domains [136]. Based on this, the Bcl-2 family is divided into three subgroups: anti-apoptotic proteins (Bcl-2, Bcl-XL, Bcl-W, Mcl-1 and Bfl-1), pro-apoptotic proteins (Bak, Bax, Bok) and proteins that act as activators and sensitizers (Bim, Bid, Bad, Noxa, Puma, Bmf, Bik, Hrk) [137,138]. Researchers showed that the overexpressed Bcl-2 in tumors seriously abrogated the killing effects of CD8^+^ CTLs and NK cells on tumor cells [139]. CTLs and NK cells destroy tumor cells by releasing perforin and granzyme-B, as well as interacting with death receptors (such as tumor necrosis factor-related apoptosis-inducing ligand [TRAIL]) on the cell surface. These functions can be disabled by the Bcl-2 protein [140]. Two small-molecule Bcl-2 inhibitors, HA14-1 and ABT-737, were found to improve the in vitro cytotoxicity of CTLs and NK cells to the lymphoma and melanoma cells, which might be attributed to sensitizing tumor cells to perforin and granzyme-B with drug treatments [140,141]. Furthermore, ABT-737 showed strong synergism with DC-based vaccines on a CT26 colon carcinoma model, indicating the immuno-sensitization effect of Bcl-2 inhibitors [140,141]. In addition to tumor cells, the inhibition of Bcl-2 could also modulate Tregs, which was exemplified by pan Bcl-2 inhibitor GX15-070 (GX15) [142]. In comparison with effector T lymphocytes, Tregs were more vulnerable to the treatment of GX15 [142,143]. This enables GX15 to specifically induce apoptosis of intra-tumoral Tregs while having a minor impact on CTLs [143,144]. Thus, treatment of GX15 resulted in downregulated expression of Foxp3 and CTLA-4, elevated ratio of CD8^+^ T-cells to Tregs, and reinforced vaccine-mediated immune response [142,144]. 

**Figure 5:**
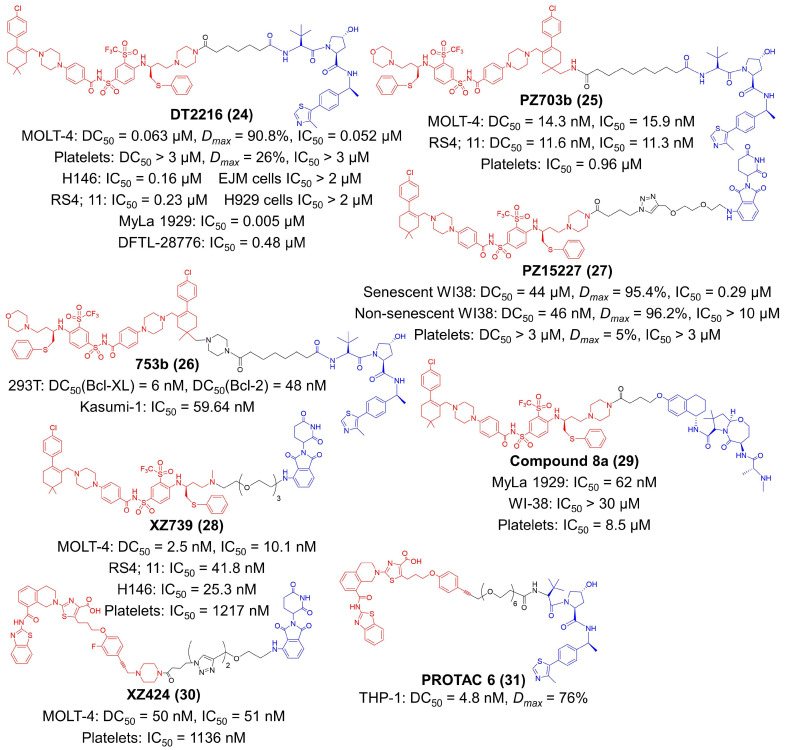
The chemical structures of representative PROTACs target the Bcl-2 family. DC_50_—half-maximal degradation concentration; *Dmax*—maximum degradation; IC_50_—half-maximal inhibition concentration.

Although inhibitors of the Bcl-2 protein family have extensive potential in tumor immunotherapy, their application was mainly restricted to hematological malignancies [145]. The therapeutic outcomes of Bcl-2 inhibitors on solid tumors are still limited [119,126]. That was because solid tumors predominantly relied on B-cell lymphoma extra-large (Bcl-XL) for survival, but most inhibitors acted widely on multiple proteins in the Bcl-2 family or selectively on the Bcl-2 protein [64,145]. The inhibitor of specific subtypes such as Bcl-XL protein might achieve satisfactory tumor therapy. However, the administration of Bcl-XL inhibitors might cause thrombocytopenia in a dose-dependent manner [64,146]. To address these issues, Zhou et al. designed a series of Bcl-XL PROTACs by linking ABT263 (a Bcl-2 and Bcl-XL dual inhibitor) to a VHL ligand [146]. Among these compounds, DT2216 was screened out due to its high Bcl-XL degradation efficiency (Figure 5) [145,146]. They identified that the VHL E3 ligase was minimally expressed in platelets; thus, the toxicity of the Bcl-XL inhibitor to platelets could be avoided [64]. DT2216 selectively degraded Bcl-XL in MOLT-4 T-cell acute lymphoblastic leukemia (T-ALL) cells (DC_50_ = 0.063 μM, *Dmax* = 90.8%), while human platelets (DC_50_ > 3 μM, *Dmax* = 26%) are less affected [64]. DT2216 binds to Bcl-XL and Bcl-2 but selectively degrades Bcl-XL. In vivo studies revealed that DT2216 had a very broad antitumor spectrum [64]. It potently suppressed the expansion of several xenograft tumors such as non-small cell lung cancer, colorectal cancer and pancreatic cancer, as well as hematologic malignancy such as T lymphoblastic leukemia [145,147,148]. They found that the VHL ligand can also be attached to the methyl groups on the cyclohexene ring of ABT263, therefore obtaining PZ703b as another novel PROTAC compound (Figure 5) [65]. PZ703b selectively degraded Bcl-XL (DC_50_ = 14.3 nM in MOLT-4 cells, DC_50_ = 11.6 nM in RS4; 11 cells), and also inhibit the activity of Bcl-2 protein by forming stable complexes with it [65]. By altering the length of the linker, Zhou’s group further converted PZ703b into a dual degrader for Bcl-XL and Bcl-2, 753b [66,149]. 753b effectively degraded both Bcl-XL (DC_50_ = 6 nM) and Bcl-2 (DC_50_ = 48 nM) in 293 T cells and exerted considerable therapeutic effect against Kasumi-1 AML cells (IC_50_ = 59.64 nM) [66]. Since the CRBN E3 ligase is also poorly expressed on platelets, CRBN-recruiting ligand pomalidomide was employed to design ABT263-based PROTAC compounds (Figure 5) [67]. The lead compound PZ15227 eliminated senescent cells (DC_50_ = 44 μM, *Dmax* = 95.4%, IC_50_ = 0.29 μM) while did not cause thrombocytopenia [67]. Immunological investigation validated that PZ15227 and DT2216 were able to alleviate the suppressive effect of tumor-infiltrated Tregs by degrading Bcl-XL [68]. They found degradation of Bcl-XL resulted in apoptosis of Tregs and activation of CD8^+^ T-cells, noting that small-molecule PROTACs for Bcl-XL can be promising adjuvant agents in tumor immunotherapy [68,150]. After slight modification of the warhead to Bcl-XL, XZ739 was reported by this group as another CRBN-dependent degrader (Figure 5) [69]. XZ739 is very potent in degrading Bcl-XL and killing MOLT-4 cells (DC_50_ = 2.5 nM, IC_50_ = 10.1 nM) with low platelets toxicity (IC_50_ = 1217 nM) [69].

Long-lasting treatment of CRBN- and VHL-based PROTACs has been observed to acquire drug resistance [70]. Therefore, Zhou et al. seek to utilize cIAP E3 ligases for protein degradation [70]. They reported Compound **8a** as a potent and selective Bcl-XL degrader in CRBN low-expressing MyLa 1929 malignant T-cell lymphoma cells (IC_50_ = 62 nM) (Figure 5) [70]. Compound **8a** leveraged ABT263 as the warhead to Bcl-XL, and its cIAP ligand was derived from LCL161, a novel antagonist against cIAP. A-1155463 is a selective Bcl-XL inhibitor, which offers an ideal option for the discovery of Bcl-XL PROTACs [71]. Zheng et al. synthesized XZ424 using A-1155463 derivative as the warhead towards Bcl-XL and pomalidomide as the CRBN ligand (Figure 5) [71]. The DC_50_ and IC_50_ value of XZ424 is 50 nM and 51 nM, respectively, in MOLT-4 cells [71]. Chung et al. reported PROTAC 6 as a high affinity degrader of Bcl-XL (IC_50_ = 0.6 nM) in a VHL-dependent mechanism (Figure 5) [72]. Structurally, the Bcl-XL ligand of PROTAC 6 was also derived from A-1155463 with modification. PROTAC 6 had a DC_50_ value of 4.8 nM and a *Dmax* of 76% against THP-1 cells [72]. 

## 4. Small-Molecule PROTACs Targeting Multifunctional Immuno-Modulating Signals 

### 4.1. Small Molecular PROTACs Targeting STAT3

Signal transducer and activator of transcription 3 (STAT3) pathway are transiently activated in normal cells, while in malignant disease, it is sustainedly overactivated by aberrant upstream receptors [151,152,153]. Tumor-promoting factors such as interleukin-6 (IL-6), IL-10, IL-11, vascular endothelial growth factor (VEGF), fibroblast growth factor (FGF), and platelet-derived growth factor (PDGF) bind to their corresponding receptors, stimulating the phosphorylation of Janus kinases (JAK), receptor tyrosine kinases (such as EGFR) and non-receptor tyrosine kinases (such as breakpoint cluster region-abelson [Bcr-Abl]) [152,154]. Subsequently, these extracellular signals are transduced into the nucleus by STAT3 protein with a cascade of recruitment, phosphorylation, dimerization and translocation, transcriptionally modifying the survival, proliferation, and metastasis of tumor cells [152,155]. Moreover, accumulating experimental results indicated that STAT3 is hyperactivated in both tumor cells and tumor-associated immune cells, which consolidates immunosuppression [151,155]. For tumor cells, STAT3 decreases the production of immuno-stimulatory cytokines such as interferon-γ (IFN-γ) and tumor necrosis factor-α (TNF-α), along with elevated expression of immuno-exhausting cytokines (IL-6, IL-10, transforming growth factor-β [TGF-β], and VEGF) [155,156]. For tumor-infiltrated immune cells, STAT3 favors colonization of Tregs and polarization of macrophages towards the M2 phenotype while restricting antigen presentation by DCs and the cytotoxic effects of CD8^+^ T-cells and NK cells [155]. Overall, STAT3 induces specific and typical alternations in diverse cells, seriously abrogating antitumor immunity. 

**Figure 6:**
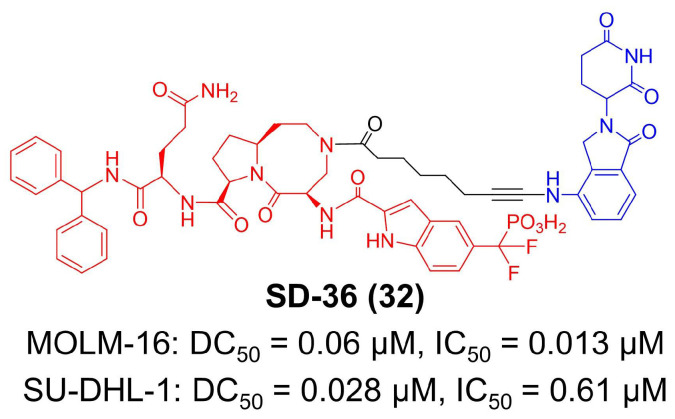
The chemical structure of the first PROTAC targeting STAT3. DC_50_—half-maximal degradation concentration; IC_50_—half-maximal inhibition concentration.

Nowadays, several studies have identified immuno-amplification effects in STAT3 inhibitors [151,153,157]. Except for tumor growth inhibition, monotherapies of STAT3 small-molecule inhibitors are found to decrease immunosuppressive macrophages and Tregs, increase cytolytic CD8^+^ T-cells and elevate the secretion of proinflammatory cytokines and chemokines [156,158,159]. Combination therapies with PD-L1 blockade or STING agonism also revealed the significance of reshaping the tumor microenvironment by STAT3 inhibition [160,161]. As a leading technology, PROTAC is supposed to further broaden and enhance the immune effects of STAT3 inhibitors. In 2019, Wang et al. reported the first small-molecule degrader for STAT3 (Figure 6) [73]. They developed SI-109 as an inhibitor of the SHP2 domain in STAT3 protein and then employed lenalidomide as the CRBN ligand, obtaining SD-36 as a nanomolar-level degrader for STAT3 [73]. In in vitro and in vivo studies, SD-36 completely and selectively induces STAT3 degradation with minor effects on other proteins in MOLM-16 (DC_50_ = 0.06 μM, IC_50_ = 0.013 μM) and SU-DHL-1 (DC_50_ = 0.028 μM, IC_50_ = 0.61 μM) cell lines [73,74]. Regarding therapeutic outcomes, SD-36 achieves long-lasting tumor repression in various xenograft mouse models [74].

### 4.2. Small-Molecule PROTACs Targeting MAPKs

The Mitogen-activated protein kinases (MAPKs) family constitutes three parallel MAPK signals, the extracellular signal-regulated kinase (ERK) pathway c-Jun N-terminal kinase (JNK) pathway and the p38 MAPK pathway [162]. MAPK pathways exert pleiotropic effects on both tumor cells and tumor-associated myeloid cells [162,163]. For tumor cells, Jong et al. demonstrated that epidermal growth factor (EGF)-dependent activation of MAPK contributed to PD-L1 expression in lung adenocarcinoma, and inhibitors of EGFR and MAPK kinase (MEK) were able to reverse this effect [164]. As for antigen-presenting cells (APCs), Yi et al. discovered that upregulated p38 MAPK pathway hampered the stimulation of antigen-pulsed DCs to T lymphocytes [165]. Hence, inhibition of p38 MAPK by multiple means improved the potency of DCs to activate antigen-specific effector T lymphocytes for tumor immunotherapy [165]. As for T lymphocytes, Lio et al. revealed that activation of the Ras/MAPK pathway is highly associated with the poor infiltration of T lymphocytes in breast tumors [166]. MEK inhibition with trametinib primed tumor immunogenicity via T-cell recruiting and achieved potent tumor suppression in combination with 4-1BB and OX-40 antibodies [166]. Mukherji et al. reported that JNK inhibitor SP600125 rescued CTLs from activation-induced cell death (AICD) triggered by vaccine epitope in vitro [167]. Similar results were observed by Mellman and co-workers in vivo studies [168]. They found that MEK inhibition prevented intra-tumoral CD8^+^ T-cells from TCR-stimulated death without affecting their cytolytic activity, and MEK inhibitor G-38963 exhibited a synergistic effect with anti-PD-L1 therapy on tumor regression [168]. 

**Figure 7:**
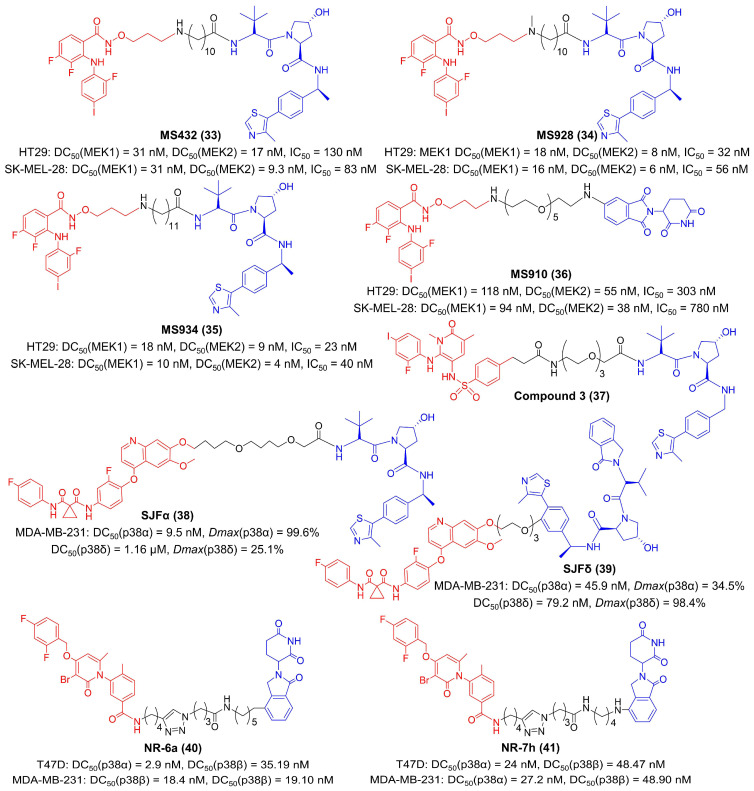
The chemical structures of representative PROTACs targeting MAPK. DC_50_—half-maximal degradation concentration; *Dmax*—maximum degradation; IC_50_—half-maximal inhibition concentration.

In 2019, Jin et al. developed the first PROTAC compound, MS432, for MEK degradation by coupling a VHL ligand to the MEK inhibitor PD0325901 (Figure 7) [75,169]. MS432 potently and specifically degraded MEK1 and MEK2 inHT29 (MEK1 DC_50_ = 31 nM, MEK2 DC_50_ = 17 nM, IC_50_ = 130 nM) and SK-MEL-28 (MEK1 DC_50_ = 31 nM, MEK2 DC_50_ = 9.3 nM, IC_50_ = 83 nM) cells [75]. One year later, they described two improved VHL-based degraders for MEK, MS928 and MS934, as well as the first CRBN-based MEK degrader MS910 (Figure 7), as a result of extensive structure-activity relationships (SAR) studies [76]. These three compounds had the same MEK warhead as previously reported MS432 and inhibited the activities of both MEK1 and MEK2 in millimicromole levels [76]. Their results revealed that compounds with longer linkers were more potent in MEK degradation and ERK phosphorylation suppression [75,76,169]. Perry et al. offered another paradigm for MEK degraders (Figure 7) [77]. They suggested that an arylsulfonamide structure was a suitable scaffold to attach to the E3 ligase ligand for MEK degradation and developed compound **3** as an effective MEK1 degrader (IC_50_ = 0.1 μM) to inhibit tumor proliferation [77]. 

There are four isoforms (α, β, γ, δ) in the p38 MAPK family, which have diverse roles in diseases. Isoform-selective therapeutics may improve the accuracy of p38 inhibition. Focusing on this issue, Crews et al. developed SJFα and SJFδ, which selectively degraded p38α and p38δ, respectively (Figure 7) [78]. Both these two compounds utilized foretinib as the p38 ligand, but the length of the linker and the attachment site to the VHL-recruiting ligand were different [78]. SJFα with a 13-atom linker and an amide attachment specifically degraded p38α (DC_50_(p38α) = 9.5 nM, *Dmax*(p38α) = 99.6%; DC_50_(p38δ) = 1.16 μM, *Dmax*(p38δ) = 25.1%), but SJFδ with a 10-atom linker and a phenyl attachment specifically degraded p38δ (DC_50_(p38α) = 45.9 nM, *Dmax*(p38α) = 34.5%; DC_50_(p38δ) = 79.2 nM, *Dmax*(p38δ) = 98.4%) in MDA-MB-231 cells [78]. Nebreda et al. found that optimizing the length and composition of the linkers was an effective strategy for isoform-selective p38 degradation [76]. They synthesized a library of compounds with PH-797804 as p38 warheads and thalidomide analogues as CRBN-recruiting ligands (Figure 7) [79]. The results identified that compound NR-6a and compound NR-7h exclusively degraded both p38α and p38β without affecting other kinases in T47D cells (DC_50_(p38α) = 2.9 nM and DC50(p38β) = 35.19 nM for NR-6a; DC_50_(p38α) = 24 nM and DC_50_(p38β) = 48.47 nM for NR-7h) and MDA-MB-231 cells (DC_50_(p38α) = 18.4 nM and DC_50_(p38β) = 19.1 nM for NR-6a; DC_50_(p38α) = 27.2 nM and DC_50_(p38β) = 48.9 nM for NR-7h) [79]. Although having not been reported, the aforementioned PROTACs with better tumor suppression efficiency than parent inhibitors are very promising in the field of tumor immunotherapy, which can be strongly supported by the proximate relationship between MAPK inhibition and immune activation [162,163,164,165,166,167,168].

## 5. The “Blue Ocean”: Unexploited and Less-Developed Targets for PROTAC-Based Immune Intervention

### 5.1. CD47-SIRPα Interaction

CD47 is a well-documented “don’t eat me” signal which interacts with the signal regulatory protein alpha (SIRPα) on the surface of macrophages, therefore protecting tumor cells against macrophagic phagocytosis [170]. Since macrophages are the key regulators in both innate and adaptive antitumor immunity, blockade of CD47-SIRPα interaction facilitates tumor clearance, as well as the engulfment and presentation of tumor-specific antigen mediated by macrophages [170]. Canonical CD47-SIRPα targeted therapeutics are antibodies, but small molecular inhibitors that target CD47-SIRPα binding and CD47 expression have emerged as better choices due to their preferable tumor accessibility [170,171] even though CD47 is also expressed on erythrocytes; thus systematic administration of CD47 inhibitor may cause anemia as a severe side effect [172]. PROTAC technology has been demonstrated to achieve cell/tissue selectivity and relieve toxicity over conventional small molecular inhibitors in studies for protein downregulation [172]. Accordingly, it is feasible to leverage the difference in E3 ubiquitin ligase expression between erythrocytes and tumor cells to avoid hematological adverse reactions of CD47-SIRPα blockade [172]. In addition, E3 ligase-dependent ubiquitination has been well-validated in the regulation of the CD47-SIRPα axis, which strongly consolidates the rationality of PROTAC application in this signal [172].

### 5.2. Foxp3

Forkhead box P3 (Foxp3) is a characteristic transcription factor expressed in Tregs [173,174]. Tregs function as an immunological “brake”, impeding the activation of CTLs [174]. Mounting evidence revealed that elimination of intra-tumoral Tregs could enhance antitumor immune response, wherein Foxp3 is a promising target due to its regulatory role in Tregs [68,142,150,174]. Several agents have been developed to inhibit the dimerization and post-translational modification of Foxp3 [173]. However, most compounds are designed for enzyme inhibition instead of directly targeting Foxp3 protein [173,174]. PROTAC technology targeting Foxp3 degradation in Tregs might be a preferable candidate.

### 5.3. COX-1/2

**Figure 8:**
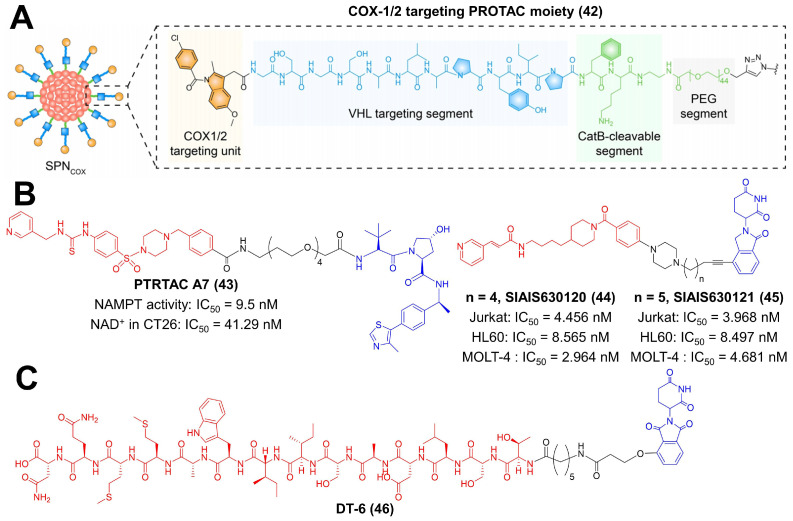
The chemical structure of representative PROTACs targeting COX-1/2, NAMPT, and TGF-β1. (**A**) The chemical structure of COX-1/2 targeting PROTAC moiety in nanomedicine. (**B**) The chemical structure of representative PROTACs targeting NAMPT. (**C**) The chemical structure of representative PROTACs targeting TGF-β1. IC_50_—half-maximal inhibition concentration.

Tumor cells tend to create an inflammatory microenvironment for their development, wherein cyclooxygenase 1/2 (COX-1/2) acts as the key regulator [175,176]. COX-1/2 is highly upregulated within tumor tissues, resulting in immune suppression [175,177]. Pu et al. constructed a smart nanomedicine loaded with a COX-1/2 targeting PROTAC moiety (Figure 8A) [80]. Indomethacin was chosen as the COX-1/2 binding unit, and it was directly connected to GSGSALAPYIP peptide, which acted as the VHL binding segment [80]. After releasing in response to cathepsin B overexpressed in the tumor, the PROTAC moiety persistently induced COX-1/2 degradation via VHL E3 ligase and depleted the metabolite of COX, prostaglandin E2 (PGE 2) [80]. PGE2 is an activator for immune suppressive cells (MDSCs, Tregs and M2 macrophage); thus, the PROTAC-mediated COX-1/2 degradation reprogrammed TME favoring tumor inhibition [80,175,177].

### 5.4. NAMPT

Recent studies demonstrated that the extracellular secreted nicotinamide mononucleotide adenylyl transferase (NAMPT) exerted cytokine-like activity to promote the expansion of MDSCs [81,178]. To reverse this effect, Sheng et al. designed a PROTAC compound to degrade intracellular NAMPT and reduce its amount in TME (Figure 8B) [81]. They prepared a series of compounds using their previously discovered NAMPT inhibitor MS7 and a VHL ligand and identified PROTAC A7 as a potent NAMPT degrader [81]. The IC_50_ value of PROTAC A7 against the catalytic activity of NAMPT was 9.5 nM [81]. In in vivo studies, treatment of PROTAC A7 inhibited the intra-tumoral infiltration of MDSCs and boosted antitumor immunity [81]. Fan et al. also reported two effective degraders for NAMPT (Figure 8B) [82]. They selected FK866 (NAMPT-specific inhibitor) as the ligand to target the protein and lenalidomide as the CRBN-recruiting group, respectively [82]. SIAIS630120 and SIAIS630121 adopted an extended piperazine group, and five (SIAIS630120) or six (SIAIS630121) atom alkyl linkers were successfully synthesized [82]. Both compounds performed superiority in protein degradation and hematological tumor cell killing than their parent inhibitor FK886 (SIAIS630120: IC_50_(Jurkat) = 4.456 nM, IC_50_(HL60) = 8.565 nM, IC_50_(MOLT-4) = 2.964 nM; SIAIS630121: IC_50_(Jurkat) = 3.968 nM, IC_50_(HL60) = 8.497 nM, IC_50_(MOLT-4) = 4.681 nM).

### 5.5. TGF-β1

Transforming growth factor-β1 (TGF-β1) is a multifunctional cytokine involved in angiogenesis, epithelial to mesenchymal transition (EMT) and immune escape of tumor cells [83,175,179]. Canonical therapeutics for TGF-β1 blocking are antisense oligonucleotides and antibodies. However, their therapeutic outcomes remain unsatisfactory, which is due to insufficient drug delivery, immune-associated side effects and drug resistance. Bu et al. reported a PROTAC compound to modulate TGF-β1 in a novel mechanism (Figure 8C) [83]. Peptide P144 (TSLDASIIWAMMQN) was employed as the binding moiety to TGF-β1, and thalidomide was adopted to induce TGF-β1 degradation by the CRBN E3 ligase [83]. Among synthesized compounds, DT-6, with the longest linker length, exhibited the best TGF-β1 degradation efficiency [83]. DT-6 primarily deprived macrophages of M2 polarization and subsequently abrogated their pro-metastatic capability via reducing the TGF-β1 secretion [83].

## 6. Conclusions and Outlook

In summary, the therapeutic targets available for PROTAC strategies are far more than checkpoint proteins. Since tumor immunotherapy is a multifactorial event, proteins in tumor cells, immune cells and tumor milieu can all be degraded by this novel technology. Future studies should develop more PROTAC molecules with preferable drug-like properties and expand their potency in immune modulation, while the following issues should be carefully taken into account: (i) More than “degrader”, PROTAC molecule can serve as an “intelligent switch” to program immune cells, which is exemplified by Park and co-workers [180,181]; (ii) certain targets had synchronous functions; therefore PROTAC molecules that simultaneously degraded two or more proteins are very promising [182,183]; (iii) E3 ligases CRBN and VHL are immuno-oncologic targets which can be degraded by homo-PROTAC strategies [184,185,186]; (iv) novel TPD strategies such as AUTAC, AbTAC, LYTAC and molecule glues have been developed to target extracellular and membrane proteins [187,188]; (v) advanced drug delivery systems (DDSs) including nanoparticles, polymetric micelles and liposomes can be leveraged to improve drug efficiency [189,190].

## Figures and Tables

**Table 1:** The summary and comparison of small-molecule PROTACs for tumor immunotherapy.

PROTAC	POI Ligand	E3 Ligase	Target	Disease Models	References
P22 (1)	Resorcinol diphenyl ether	CRBN	PD-L1	Hep3B/OS-8/hPD-Ll cells	[38]
21a (2)	BMS-37			MC38 cells, MC38 xenograft model	[39]
SHP-D26 (3)	SHP099	VHL	SHP2	KYSE520 cells, MV4: 11 AML cells	[40]
SP4 (4)		CRBN		Hela cells	[41]
ZB-S-29 (5)	TNO155 analogue	CRBN	SHP2	MV4; 11 cells	[42]
dBET1 (6)	JQ1	CRBN	BRD2/3/4	SUM149 cells, leukemia mice model	[43]
MZ1 (7)		VHL		HCT116 HNSCC cells	[44,45]
ARV-771 (8)	JQ1	VHL		22RV1 CRPC cells, 22RV1 CRPC xenograft model	[46]
ARV-825 (9)	OTX015	CRBN		Namalwa cells, Ramous cells, CA-46 cells, DAUDI cells, SKO-007(J3) human MM cells, HCT116 cells	[47,48]
BET-d246 (10)	HJB97	CRBN		MDA-MB-468 cells, WHIM24 PDX model, MDA-MB-453/231/468 xenograft models, HCT116 cells	[49,50]
BET-d260 (11)				RS4;11 cells, MOLM-13 cells, HCT116 cells, HCT116 xenograft model, CRC PDX model, CT26 xenograft model	[50,51]
QCA570 (12)	1, 4-Oxazeopine	CRBN		MV4; 11 cells, MOLM-13 cells, RS4: 11 cells; RS4: 11 xenograft model, MV4: 11 xenograft model	[52]
Compound 2c (13)	Epacadostat	CRBN	IDO1	Hela cells	[53]
IDO-targeting PROTRAC peptide (14)	NLG919	VHL		4T1 cells, 4T1 xenograft model	[54]
PROTAC 4 (15)	Benzamide-based structure	VHL	HDAC1/2/3	HCT166 cells	[55]
Compound 9c (16)	Vorinostat analogue	CRBN	HDAC6	MCF-7 cells	[56]
Compound 12d (17)	Nexturastat A	CRBN		MM1S human cells,	[57]
Compound 3j (18)		VHL		MM1S cells, 4935 murine cells	[58]
NP8 (19)		CRBN		MM1S cells	[59]
NH2 (20)				MM1S cells	[60]
HD-TAC7 (21)	CI994	CRBN	HDAC3	Raw 264.7 murine macrophages	[61]
XZ9002 (22)	SR33558	VHL		MDA-MB-231 cells	[62]
Compound 12 (23)	Sirtuin rearranging ligand	CRBN	Sirt2	Hela cells	[63]
DT2216 (24)	ABT263	VHL	Bcl-XL	MOLT-4 T-ALL cells, H146 cells, RS4; 11 cells, Myla 1929 cells, DFTL-28776 cells, MOLT-4 T-ALL xenograft model, H146 SCLC xenograft model, MDA-MB-231 xenograft model, CUL76 T-ALL PDX model	[64]
PZ703b (25)			Bcl-XL	MOLT-4 cells, RS4; 11 cells	[65]
753b (26)			Bcl-XL/2	293T cells, Kasumi-1 AML cells	[66]
PZ15227 (27)		CRBN	BCL-XL	W138 cells, aged mice model, Renca cells, Renca xenograft model	[67,68]
XZ739 (28)	ABT263 analogue	CRBN	Bcl-XL	MOLT-4 cells	[69]
Compound 8a (29)	ABT263	cIAP		MyLa 1929 malignant T-cell lymphoma cells	[70]
XZ424 (30)	A-1155463 derivative	CRBN		MOLM-4 cells	[71]
PROTAC 6 (31)		VHL		THP-1 cells	[72]
SD-36 (32)	SI-109	CRBN	STAT3	MOLM-16 cells, SU-DHL-1 cells, MOLM-16 xenograft model, SU-DHL-1 xenograft model	[73,74]
MS432 (33)	PD0325901	VHL	MEK1/2	HT29 cells, SK-MEL-28 cells	[75]
MS928 (34)		VHL			[76]
MS934 (35)		VHL			
MS910 (36)		CRBN			
Compound 3 (37)	Arylsulfonamide	VHL	MEK1	A375 cells	[77]
SJFα (38)	Foretinib	VHL	p38α	MDA-MB-231 cells	[78]
SJFδ (39)			p38δ		
NR-6a (40)	PH-797804	CRBN	p38α/β	T47D cells, MDA-MB-231 cells	[79]
NR-7h (41)					
COX-1/2 targeting PROTAC moiety (42)	Indomethacin	VHL	COX-1/2	4T1 cells, 4T1 xenograft model	[80]
PROTAC A7 (43)	MS7	VHL	NAMPT	CT26 cells	[81]
SIAIS630120 (44)	FK866	CRBN		Jurkat cells, HL60 cells, MOLT-4 cells	[82]
SIAIS630121 (45)					
DT-6 (46)	P144 (TSLDASIIWAMMQN)	CRBN	TGF-β1	A549 cells, U87 cells, MCF-7 cells, HepG2 cells, THP-1 cells, BV2 cells	[83]

AML: acute myeloid leukemia, HNSCC— head and neck squamous cell carcinoma, CRPC: castration-resistant prostate cancer, MM—multiple myeloma, PDX—patient-derived tumor xenograft, T-ALL—T-cell acute lymphoblastic leukemia, SCLC—small cell lung cancer.

## Data Availability

Not applicable.

## References

[1] Osipov A., Saung M.T., Zheng L., Murphy A.G. (2019). Small molecule immunomodulation: The tumor microenvironment and overcoming immune escape. J. Immunother. Cancer.

[2] Van der Zanden S.Y., Luimstra J.J., Neefjes J., Borst J., Ovaa H. (2020). Opportunities for Small Molecules in Cancer Immunotherapy. Trends Immunol..

[3] Li Y., Yang L., Xu X., Li M., Zhang Y., Lin Q., Gong T., Sun X., Zhang Z., Zhang L. (2021). Multifunctional Size-Expandable Nanomedicines Enhance Tumor Accumulation and Penetration for Synergistic Chemo-Photothermal Therapy. ACS Appl. Mater. Interfaces.

[4] Weissleder R., Pittet M.J. (2020). The expanding landscape of inflammatory cells affecting cancer therapy. Nat. Biomed. Eng..

[5] Ding C., Song Z., Shen A., Chen T., Zhang A. (2020). Small molecules targeting the innate immune cGAS–STING–TBK1 signaling pathway. Acta Pharm. Sin. B.

[6] Hofer F., Di Sario G., Musiu C., Sartoris S., de Sanctis F., Ugel S. (2021). A Complex Metabolic Network Confers Immunosuppressive Functions to Myeloid-Derived Suppressor Cells (MDSCs) within the Tumour Microenvironment. Cells.

[7] Xiang Y., Chen L., Li L., Huang Y. (2020). Restoration and Enhancement of Immunogenic Cell Death of Cisplatin by Coadministration with Digoxin and Conjugation to HPMA Copolymer. ACS Appl. Mater. Interfaces.

[8] Ma S., Qin L., Wang X., Wang W., Li J., Wang H., Li H., Cai X., Yang Y., Qu M. (2022). The Expression of VISTA on CD4+ T Cells Associate with Poor Prognosis and Immune Status in Non-small Cell Lung Cancer Patients. Bosn. J. Basic Med. Sci..

[9] Cheng B., Yuan W.-E., Su J., Liu Y., Chen J. (2018). Recent advances in small molecule based cancer immunotherapy. Eur. J. Med. Chem..

[10] Kotzner L., Huck B., Garg S., Urbahns K. (2020). Small molecules-Giant leaps for immuno-oncology. Prog. Med. Chem..

[11] Chen L., Liu C., Xiang Y., Lyu J., Zhou Z., Gong T., Gao H., Li L., Huang Y. (2022). Exocytosis blockade of endoplasmic reticulum-targeted nanoparticle enhances immunotherapy. Nano Today.

[12] Xiang Y., Chen L., Liu C., Yi X., Li L., Huang Y. (2022). Redirecting Chemotherapeutics to the Endoplasmic Reticulum Increases Tumor Immunogenicity and Potentiates Anti-PD-L1 Therapy. Small.

[13] Zhang J., Zhang Y., Qu B., Yang H., Hu S., Dong X. (2021). If small molecules immunotherapy comes, can the prime be far behind?. Eur. J. Med. Chem..

[14] Song Z., Zhang A. (2019). Small-Molecule Immuno-Oncology Therapy: Advances, Challenges and New Directions. Curr. Top. Med. Chem..

[15] Toogood P.L. (2018). Small molecule immuno-oncology therapeutic agents. Bioorg. Med. Chem. Lett..

[16] Bekes M., Langley D.R., Crews C.M. (2022). PROTAC targeted protein degraders: The past is prologue. Nat. Rev. Drug Discov..

[17] Mullard A. (2021). Targeted protein degraders crowd into the clinic. Nat. Rev. Drug Discov..

[18] Li X., Pu W., Zheng Q., Ai M., Chen S., Peng Y. (2022). Proteolysis-targeting chimeras (PROTACs) in cancer therapy. Mol. Cancer.

[19] Bond M.J., Crews C.M. (2021). Proteolysis targeting chimeras (PROTACs) come of age: Entering the third decade of targeted protein degradation. RSC Chem. Biol..

[20] Li K., Crews C.M. (2022). PROTACs: Past, present and future. Chem. Soc. Rev..

[21] Lu X., Smaill J.B., Ding K. (2020). New Promise and Opportunities for Allosteric Kinase Inhibitors. Angew. Chem. Int. Ed..

[22] Roskoski R. (2019). Properties of FDA-approved small molecule protein kinase inhibitors. Pharmacol. Res..

[23] Ferguson F.M., Gray N.S. (2018). Kinase inhibitors: The road ahead. Nat. Rev. Drug Discov..

[24] Xiang S., Song S., Tang H., Smaill J.B., Wang A., Xie H., Lu X. (2021). TANK-binding kinase 1 (TBK1): An emerging therapeutic target for drug discovery. Drug Discov. Today.

[25] He M., Lv W., Rao Y. (2021). Opportunities and Challenges of Small Molecule Induced Targeted Protein Degradation. Front. Cell Dev. Biol..

[26] Wang C., Zheng C., Wang H., Zhang L., Liu Z., Xu P. (2022). The state of the art of PROTAC technologies for drug discovery. Eur. J. Med. Chem..

[27] Ishida T., Ciulli A. (2021). E3 Ligase Ligands for PROTACs: How They Were Found and How to Discover New Ones. SLAS Discov. Adv. Sci. Drug Discov..

[28] Cao C., He M., Wang L., He Y., Rao Y. (2022). Chemistries of bifunctional PROTAC degraders. Chem. Soc. Rev..

[29] He S., Dong G., Cheng J., Wu Y., Sheng C. (2022). Strategies for designing proteolysis targeting chimaeras (PROTACs). Med. Res. Rev..

[30] Sosič I., Bricelj A., Steinebach C. (2022). E3 ligase ligand chemistries: From building blocks to protein degraders. Chem. Soc. Rev..

[31] Chen Y., Jin J. (2020). The application of ubiquitin ligases in the PROTAC drug design. Acta Biochim. Biophys. Sin..

[32] Lin J., Jin J., Shen Y., Zhang L., Gong G., Bian H., Chen H., Nagle D.G., Wu Y., Zhang W. (2021). Emerging protein degradation strategies: Expanding the scope to extracellular and membrane proteins. Theranostics.

[33] Xu J., Brosseau J.P., Shi H. (2020). Targeted degradation of immune checkpoint proteins: Emerging strategies for cancer immunotherapy. Oncogene.

[34] Wang E.A.Y., Deng S., Xu J. (2020). Proteasomal and lysosomal degradation for specific and durable suppression of immunotherapeutic targets. Cancer Biol. Med..

[35] Irvine D.J., Dane E.L. (2020). Enhancing cancer immunotherapy with nanomedicine. Nat. Rev. Immunol..

[36] Liu C., Li L., Lyu J., Xiang Y., Chen L., Zhou Z., Huang Y. (2022). Split bullets loaded nanoparticles for amplified immunotherapy. J. Control. Release.

[37] He M., Cao C., Ni Z., Liu Y., Song P., Hao S., He Y., Sun X., Rao Y. (2022). PROTACs: Great opportunities for academia and industry (an update from 2020 to 2021). Signal Transduct. Target. Ther..

[38] Cheng B., Ren Y., Cao H., Chen J. (2020). Discovery of novel resorcinol diphenyl ether-based PROTAC-like molecules as dual inhibitors and degraders of PD-L1. Eur. J. Med. Chem..

[39] Wang Y., Zhou Y., Cao S., Sun Y., Dong Z., Li C., Wang H., Yao Y., Yu H., Song X. (2021). In vitro and in vivo degradation of programmed cell death ligand 1 (PD-L1) by a proteolysis targeting chimera (PROTAC). Bioorg. Chem..

[40] Wang M., Lu J., Wang M., Yang C.Y., Wang S. (2020). Discovery of SHP2-D26 as a First, Potent, and Effective PROTAC Degrader of SHP2 Protein. J. Med. Chem..

[41] Zheng M., Liu Y., Wu C., Yang K., Wang Q., Zhou Y., Chen L., Li H. (2021). Novel PROTACs for degradation of SHP2 protein. Bioorg. Chem..

[42] Yang X., Wang Z., Pei Y., Song N., Xu L., Feng B., Wang H., Luo X., Hu X., Qiu X. (2021). Discovery of thalidomide-based PROTAC small molecules as the highly efficient SHP2 degraders. Eur. J. Med. Chem..

[43] Winter G.E., Buckley D.L., Paulk J., Roberts J.M., Souza A., Dhe-Paganon S., Bradner J.E. (2015). Phthalimide conjugation as a strategy for in vivo target protein degradation. Science.

[44] Zengerle M., Chan K.H., Ciulli A. (2015). Selective Small Molecule Induced Degradation of the BET Bromodomain Protein BRD4. ACS Chem. Biol..

[45] Bhola N.E., Njatcha C., Hu L., Lee E.D., Ba J.V.S., Kim M., Johnson D.E., Grandis J.R. (2021). PD-L1 is upregulated via BRD2 in head and neck squamous cell carcinoma models of acquired cetuximab resistance. Head Neck.

[46] Raina K., Lu J., Qian Y., Altieri M., Gordon D., Rossi A.M.K., Wang J., Chen X., Dong H., Siu K. (2016). PROTAC-induced BET protein degradation as a therapy for castration-resistant prostate cancer. Proc. Natl. Acad. Sci. USA.

[47] Abruzzese M.P., Bilotta M.T., Fionda C., Zingoni A., Soriani A., Vulpis E., Borrelli C., Zitti B., Petrucci M.T., Ricciardi M.R. (2016). Inhibition of bromodomain and extra-terminal (BET) proteins increases NKG2D ligand MICA expression and sensitivity to NK cell-mediated cytotoxicity in multiple myeloma cells: Role of cMYC-IRF4-miR-125b interplay. J. Hematol. Oncol..

[48] Lu J., Qian Y., Altieri M., Dong H., Wang J., Raina K., Hines J., Winkler J.D., Crew A.P., Coleman K. (2015). Hijacking the E3 Ubiquitin Ligase Cereblon to Efficiently Target BRD4. Chem. Biol..

[49] Bai L., Zhou B., Yang C.-Y., Ji J., McEachern D., Przybranowski S., Jiang H., Hu J., Xu F., Zhao Y. (2017). Targeted Degradation of BET Proteins in Triple-Negative Breast Cancer. Cancer Res..

[50] Tong J., Tan X., Risnik D., Gao M., Song X., Ermine K., Shen L., Wang S., Yu J., Zhang L. (2021). BET protein degradation triggers DR5-mediated immunogenic cell death to suppress colorectal cancer and potentiate immune checkpoint blockade. Oncogene.

[51] Zhou B., Hu J., Xu F., Chen Z., Bai L., Fernandez-Salas E., Lin M., Liu L., Yang C.Y., Zhao Y. (2018). Discovery of a Small-Molecule Degrader of Bromodomain and Extra-Terminal (BET) Proteins with Picomolar Cellular Potencies and Capable of Achieving Tumor Regression. J. Med. Chem..

[52] Qin C., Hu Y., Zhou B., Fernandez-Salas E., Yang C.-Y., Liu L., McEachern D., Przybranowski S., Wang M., Stuckey J. (2018). Discovery of QCA570 as an Exceptionally Potent and Efficacious Proteolysis Targeting Chimera (PROTAC) Degrader of the Bromodomain and Extra-Terminal (BET) Proteins Capable of Inducing Complete and Durable Tumor Regression. J. Med. Chem..

[53] Hu M., Zhou W., Wang Y., Yao D., Ye T., Yao Y., Chen B., Liu G., Yang X., Wang W. (2020). Discovery of the first potent proteolysis targeting chimera (PROTAC) degrader of indoleamine 2,3-dioxygenase 1. Acta Pharm. Sin. B.

[54] Zhang C., Zeng Z., Cui D., He S., Jiang Y., Li J., Huang J., Pu K. (2021). Semiconducting polymer nano-PROTACs for activatable photo-immunometabolic cancer therapy. Nat. Commun..

[55] Smalley J.P., Adams G.E., Millard C.J., Song Y., Norris J.K.S., Schwabe J.W.R., Cowley S.M., Hodgkinson J.T. (2020). PROTAC-mediated degradation of class I histone deacetylase enzymes in corepressor complexes. Chem. Commun..

[56] Yang K., Song Y., Xie H., Wu H., Wu Y.-T., Leisten E.D., Tang W. (2018). Development of the first small molecule histone deacetylase 6 (HDAC6) degraders. Bioorg. Med. Chem. Lett..

[57] Wu H., Yang K., Zhang Z., Leisten E.D., Li Z., Xie H., Liu J., Smith K.A., Novakova Z., Barinka C. (2019). Development of Multifunctional Histone Deacetylase 6 Degraders with Potent Antimyeloma Activity. J. Med. Chem..

[58] Yang K., Wu H., Zhang Z., Leisten E.D., Nie X., Liu B., Wen Z., Zhang J., Cunningham M.D., Tang W. (2020). Development of Selective Histone Deacetylase 6 (HDAC6) Degraders Recruiting Von Hippel-Lindau (VHL) E3 Ubiquitin Ligase. ACS Med. Chem. Lett..

[59] Yang H., Lv W., He M., Deng H., Li H., Wu W., Rao Y. (2019). Plasticity in designing PROTACs for selective and potent degradation of HDAC6. Chem. Commun..

[60] An Z., Lv W., Su S., Wu W., Rao Y. (2019). Developing potent PROTACs tools for selective degradation of HDAC6 protein. Protein Cell.

[61] Cao F., de Weerd S., Chen D., Zwinderman M.R.H., van der Wouden P.E., Dekker F.J. (2020). Induced protein degradation of histone deacetylases 3 (HDAC3) by proteolysis targeting chimera (PROTAC). Eur. J. Med. Chem..

[62] Xiao Y., Wang J., Zhao L.Y., Chen X., Zheng G., Zhang X., Liao D. (2020). Discovery of histone deacetylase 3 (HDAC3)-specific PROTACs. Chem. Commun..

[63] Schiedel M., Herp D., Hammelmann S., Swyter S., Lehotzky A., Robaa D., Olah J., Ovadi J., Sippl W., Jung M. (2018). Chemically Induced Degradation of Sirtuin 2 (Sirt2) by a Proteolysis Targeting Chimera (PROTAC) Based on Sirtuin Rearranging Ligands (SirReals). J. Med. Chem..

[64] Khan S., Zhang X., Lv D., Zhang Q., He Y., Zhang P., Liu X., Thummuri D., Yuan Y., Wiegand J.S. (2019). A selective BCL-XL PROTAC degrader achieves safe and potent antitumor activity. Nat. Med..

[65] Pal P., Thummuri D., Lv D., Liu X., Zhang P., Hu W., Poddar S.K., Hua N., Khan S., Yuan Y. (2021). Discovery of a Novel BCL-XL PROTAC Degrader with Enhanced BCL-2 Inhibition. J. Med. Chem..

[66] Lv D., Pal P., Liu X., Jia Y., Thummuri D., Zhang P., Hu W., Pei J., Zhang Q., Zhou S. (2021). Development of a BCL-xL and BCL-2 dual degrader with improved anti-leukemic activity. Nat. Commun..

[67] He Y., Zhang X., Chang J., Kim H.N., Zhang P., Wang Y., Khan S., Liu X., Zhang X., Lv D. (2020). Using proteolysis-targeting chimera technology to reduce navitoclax platelet toxicity and improve its senolytic activity. Nat. Commun..

[68] Kolb R., De U., Khan S., Luo Y., Kim M.C., Yu H., Wu C., Mo J., Zhang X., Zhang P. (2021). Proteolysis-targeting chimera against BCL-XL destroys tumor-infiltrating regulatory T cells. Nat. Commun..

[69] Zhang X., Thummuri D., Liu X., Hu W., Zhang P., Khan S., Yuan Y., Zhou D., Zheng G. (2020). Discovery of PROTAC BCL-XL degraders as potent anticancer agents with low on-target platelet toxicity. Eur. J. Med. Chem..

[70] Zhang X., He Y., Zhang P., Budamagunta V., Lv D., Thummuri D., Yang Y., Pei J., Yuan Y., Zhou D. (2020). Discovery of IAP-recruiting BCL-XL PROTACs as potent degraders across multiple cancer cell lines. Eur. J. Med. Chem..

[71] Zhang X., Thummuri D., He Y., Liu X., Zhang P., Zhou D., Zheng G. (2019). Utilizing PROTAC technology to address the on-target platelet toxicity associated with inhibition of BCL-XL. Chem. Commun..

[72] Chung C.W., Dai H., Fernandez E., Tinworth C.P., Churcher I., Cryan J., Denyer J., Harling J.D., Konopacka A., Queisser M.A. (2020). Structural Insights into PROTAC-Mediated Degradation of Bcl-xL. ACS Chem. Biol..

[73] Zhou H., Bai L., Xu R., Zhao Y., Chen J., McEachern D., Chinnaswamy K., Wen B., Dai L., Kumar P. (2019). Structure-Based Discovery of SD-36 as a Potent, Selective, and Efficacious PROTAC Degrader of STAT3 Protein. J. Med. Chem..

[74] Bai L., Zhou H., Xu R., Zhao Y., Chinnaswamy K., McEachern D., Chen J., Yang C.Y., Liu Z., Wang M. (2019). A Potent and Selective Small-Molecule Degrader of STAT3 Achieves Complete Tumor Regression In Vivo. Cancer Cell.

[75] Wei J., Hu J., Wang L., Xie L., Jin M.S., Chen X., Liu J., Jin J. (2019). Discovery of a First-in-Class Mitogen-Activated Protein Kinase Kinase 1/2 Degrader. J. Med. Chem..

[76] Hu J., Wei J., Yim H., Wang L., Xie L., Jin M.S., Kabir M., Qin L., Chen X., Liu J. (2020). Potent and Selective Mitogen-Activated Protein Kinase Kinase 1/2 (MEK1/2) Heterobifunctional Small-molecule Degraders. J. Med. Chem..

[77] Vollmer S., Cunoosamy D., Lv H., Feng H., Li X., Nan Z., Yang W., Perry M.W.D. (2020). Design, Synthesis, and Biological Evaluation of MEK PROTACs. J. Med. Chem..

[78] Smith B.E., Wang S.L., Jaime-Figueroa S., Harbin A., Wang J., Hamman B.D., Crews C.M. (2019). Differential PROTAC substrate specificity dictated by orientation of recruited E3 ligase. Nat. Commun..

[79] Donoghue C., Cubillos-Rojas M., Gutierrez-Prat N., Sanchez-Zarzalejo C., Verdaguer X., Riera A., Nebreda A.R. (2020). Optimal linker length for small molecule PROTACs that selectively target p38alpha and p38beta for degradation. Eur. J. Med. Chem..

[80] Zhang C., He S., Zeng Z., Cheng P., Pu K. (2022). Smart Nano-PROTACs Reprogram Tumor Microenvironment for Activatable Photo-metabolic Cancer Immunotherapy. Angew. Chem. Int. Ed..

[81] Wu Y., Pu C., Fu Y., Dong G., Huang M., Sheng C. (2022). NAMPT-targeting PROTAC promotes antitumor immunity via suppressing myeloid-derived suppressor cell expansion. Acta Pharm. Sin. B.

[82] Zhu X., Liu H., Chen L., Cang Y., Jiang B., Yang X., Fan G. (2021). Addressing Enzymatic-Independent Tumor-Promoting Function of NAMPT via PROTAC-Mediated Degradation. bioRxiv.

[83] Feng Y., Su H., Li Y., Luo C., Xu H., Wang Y., Sun H., Wan G., Zhou B., Bu X. (2020). Degradation of intracellular TGF-beta1 by PROTACs efficiently reverses M2 macrophage induced malignant pathological events. Chem. Commun..

[84] Wu X., Meng Y., Liu L., Gong G., Zhang H., Hou Y., Liu C., Wu D., Qin M. (2021). Insights into non-peptide small-molecule inhibitors of the PD-1/PD-L1 interaction: Development and perspective. Bioorg. Med. Chem..

[85] Dai X., Gao Y., Wei W. (2021). Post-Translational Regulations of PD-L1 and PD-1: Mechanisms and Opportunities for Combined Immunotherapy. Semin. Cancer Biol..

[86] Wu Q., Jiang L., Li S.C., He Q.J., Yang B., Cao J. (2021). Small molecule inhibitors targeting the PD-1/PD-L1 signaling pathway. Acta Pharmacol. Sin..

[87] Cotton A.D., Nguyen D.P., Gramespacher J.A., Seiple I.B., Wells J.A. (2021). Development of Antibody-Based PROTACs for the Degradation of the Cell-Surface Immune Checkpoint Protein PD-L1. J. Am. Chem. Soc..

[88] Banik S.M., Pedram K., Wisnovsky S., Ahn G., Riley N.M., Bertozzi C.R. (2020). Lysosome-targeting chimaeras for degradation of extracellular proteins. Nature.

[89] Wang H., Yao H., Li C., Shi H., Lan J., Li Z., Zhang Y., Liang L., Fang J.-Y., Xu J. (2019). HIP1R targets PD-L1 to lysosomal degradation to alter T cell–mediated cytotoxicity. Nat. Chem. Biol..

[90] Yang Y., Hsu J.-M., Sun L., Chan L.-C., Li C.-W., Hsu J.L., Wei Y., Xia W., Hou J., Qiu Y. (2019). Palmitoylation stabilizes PD-L1 to promote breast tumor growth. Cell Res..

[91] Dong L., Han D., Meng X., Xu M., Zheng C., Xia Q. (2021). Activating Mutation of SHP2 Establishes a Tumorigenic Phonotype Through Cell-Autonomous and Non-Cell-Autonomous Mechanisms. Front. Cell Dev. Biol..

[92] Song Y., Wang S., Zhao M., Yang X., Yu B. (2022). Strategies Targeting Protein Tyrosine Phosphatase SHP2 for Cancer Therapy. J. Med. Chem..

[93] Yuan X., Bu H., Zhou J., Yang C.-Y., Zhang H. (2020). Recent Advances of SHP2 Inhibitors in Cancer Therapy: Current Development and Clinical Application. J. Med. Chem..

[94] Kanumuri R., Pasupuleti S.K., Burns S.S., Ramdas B., Kapur R. (2022). Targeting SHP2 phosphatase in hematological malignancies. Expert Opin. Ther. Targets.

[95] Liu Y., Yang X., Wang Y., Yang Y., Sun D., Li H., Chen L. (2021). Targeting SHP2 as a therapeutic strategy for inflammatory diseases. Eur. J. Med. Chem..

[96] Song Y., Zhao M., Zhang H., Yu B. (2022). Double-edged roles of protein tyrosine phosphatase SHP2 in cancer and its inhibitors in clinical trials. Pharmacol. Ther..

[97] Liu M., Gao S., Elhassan R.M., Hou X., Fang H. (2021). Strategies to overcome drug resistance using SHP2 inhibitors. Acta Pharm. Sin. B.

[98] Zhao M., Guo W., Wu Y., Yang C., Zhong L., Deng G., Zhu Y., Liu W., Gu Y., Lu Y. (2019). SHP2 inhibition triggers anti-tumor immunity and synergizes with PD-1 blockade. Acta Pharm. Sin. B.

[99] Liu C., Lu H., Wang H., Loo A., Zhang X., Yang G., Kowal C., Delach S., Wang Y., Goldoni S. (2021). Combinations with Allosteric SHP2 Inhibitor TNO155 to Block Receptor Tyrosine Kinase Signaling. Clin. Cancer Res..

[100] Fan Z., Tian Y., Chen Z., Liu L., Zhou Q., He J., Coleman J., Dong C., Li N., Huang J. (2020). Blocking interaction between SHP2 and PD-1 denotes a novel opportunity for developing PD-1 inhibitors. EMBO Mol. Med..

[101] Ramesh A., Kumar S., Nandi D., Kulkarni A. (2019). CSF1R- and SHP2-Inhibitor-Loaded Nanoparticles Enhance Cytotoxic Activity and Phagocytosis in Tumor-Associated Macrophages. Adv. Mater..

[102] Quintana E., Schulze C.J., Myers D.R., Choy T.J., Mordec K., Wildes D., Shifrin N.T., Belwafa A., Koltun E.S., Gill A.L. (2020). Allosteric Inhibition of SHP2 Stimulates Antitumor Immunity by Transforming the Immunosuppressive Environment. Cancer Res..

[103] Tang K., Jia Y.-N., Yu B., Liu H.-M. (2020). Medicinal chemistry strategies for the development of protein tyrosine phosphatase SHP2 inhibitors and PROTAC degraders. Eur. J. Med. Chem..

[104] Ma T., Chen Y., Yi Z.-G., Li Y.-H., Bai J., Li L.-J., Zhang L.-S. (2022). BET in hematologic tumors: Immunity, pathogenesis, clinical trials and drug combinations. Genes Dis..

[105] Wang N., Wu R., Tang D., Kang R. (2021). The BET family in immunity and disease. Signal Transduct. Target. Ther..

[106] Mori J.O., Shafran J.S., Stojanova M., Katz M.H., Gignac G.A., Wisco J.J., Heaphy C.M., Denis G.V. (2022). Novel forms of prostate cancer chemoresistance to successful androgen deprivation therapy demand new approaches: Rationale for targeting BET proteins. Prostate.

[107] Cochran A.G., Conery A.R., Sims R.J. (2019). Bromodomains: A new target class for drug development. Nat. Rev. Drug Discov..

[108] Doroshow D.B., Eder J.P., LoRusso P.M. (2017). BET inhibitors: A novel epigenetic approach. Ann. Oncol..

[109] Bechter O., Schöffski P. (2020). Make your best BET: The emerging role of BET inhibitor treatment in malignant tumors. Pharmacol. Ther..

[110] Mao W., Ghasemzadeh A., Freeman Z., Obradovic A., Chaimowitz M.G., Nirschl T.R., McKiernan E., Yegnasubramanian S., Drake C.G. (2019). Immunogenicity of prostate cancer is augmented by BET bromodomain inhibition. J. Immunother. Cancer.

[111] Andrieu G.P., Shafran J.S., Smith C.L., Belkina A.C., Casey A.N., Jafari N., Denis G.V. (2019). BET protein targeting suppresses the PD-1/PD-L1 pathway in triple-negative breast cancer and elicits anti-tumor immune response. Cancer Lett..

[112] Hao X. Overview of Tumor Immunotherapy Based on Indoleamine 2,3 Dioxygenase Inhibitors. Proceedings of the 2020 7th International Conference on Biomedical and Bioinformatics Engineering.

[113] Feng X., Liao D., Liu D., Ping A., Li Z., Bian J. (2020). Development of Indoleamine 2,3-Dioxygenase 1 Inhibitors for Cancer Therapy and Beyond: A Recent Perspective. J. Med. Chem..

[114] Tang K., Wang B., Yu B., Liu H.-M. (2022). Indoleamine 2,3-dioxygenase 1 (IDO1) inhibitors and PROTAC-based degraders for cancer therapy. Eur. J. Med. Chem..

[115] Tang K., Wu Y.-H., Song Y., Yu B. (2021). Indoleamine 2,3-dioxygenase 1 (IDO1) inhibitors in clinical trials for cancer immunotherapy. J. Hematol. Oncol..

[116] Wang C., Zhang Y., Xing D., Zhang R. (2021). PROTACs technology for targeting non-oncoproteins: Advances and perspectives. Bioorg. Chem..

[117] Zeng S., Huang W., Zheng X., Cheng L., Zhang Z., Wang J., Shen Z. (2021). Proteolysis targeting chimera (PROTAC) in drug discovery paradigm: Recent progress and future challenges. Eur. J. Med. Chem..

[118] Wang C., Zhang Y., Wang J., Xing D. (2022). VHL-based PROTACs as potential therapeutic agents: Recent progress and perspectives. Eur. J. Med. Chem..

[119] Wang K., Zhou H. (2021). Proteolysis targeting chimera (PROTAC) for epidermal growth factor receptor enhances anti-tumor immunity in non-small cell lung cancer. Drug Dev. Res..

[120] Jenke R., Ressing N., Hansen F.K., Aigner A., Buch T. (2021). Anticancer Therapy with HDAC Inhibitors: Mechanism-Based Combination Strategies and Future Perspectives. Cancers.

[121] Shanmugam G., Rakshit S., Sarkar K. (2022). HDAC inhibitors: Targets for tumor therapy, immune modulation and lung diseases. Transl. Oncol..

[122] Burke B., Eden C., Perez C., Belshoff A., Hart S., Plaza-Rojas L., Reyes M.D., Prajapati K., Voelkel-Johnson C., Henry E. (2020). Inhibition of Histone Deacetylase (HDAC) Enhances Checkpoint Blockade Efficacy by Rendering Bladder Cancer Cells Visible for T Cell-Mediated Destruction. Front. Oncol..

[123] Bretz A.C., Parnitzke U., Kronthaler K., Dreker T., Bartz R., Hermann F., Ammendola A., Wulff T., Hamm S. (2019). Domatinostat favors the immunotherapy response by modulating the tumor immune microenvironment (TIME). J. Immunother. Cancer.

[124] Zheng H., Zhao W., Yan C., Watson C.C., Massengill M., Xie M., Massengill C., Noyes D.R., Martinez G.V., Afzal R. (2016). HDAC Inhibitors Enhance T-Cell Chemokine Expression and Augment Response to PD-1 Immunotherapy in Lung Adenocarcinoma. Clin. Cancer Res..

[125] Hicks K.C., Fantini M., Donahue R.N., Schwab A., Knudson K.M., Tritsch S.R., Jochems C., Clavijo P.E., Allen C.T., Hodge J.W. (2018). Epigenetic priming of both tumor and NK cells augments antibody-dependent cellular cytotoxicity elicited by the anti-PD-L1 antibody avelumab against multiple carcinoma cell types. OncoImmunology.

[126] Chen M.C., Lin Y.C., Liao Y.H., Liou J.P., Chen C.H. (2019). MPT0G612, a Novel HDAC6 Inhibitor, Induces Apoptosis and Suppresses IFN-gamma-Induced Programmed Death-Ligand 1 in Human Colorectal Carcinoma Cells. Cancers.

[127] Christmas B.J., Rafie C.I., Hopkins A.C., Scott B.A., Ma H.S., Cruz K.A., Woolman S., Armstrong T.D., Connolly R.M., Azad N.A. (2018). Entinostat Converts Immune-Resistant Breast and Pancreatic Cancers into Checkpoint-Responsive Tumors by Reprogramming Tumor-Infiltrating MDSCs. Cancer Immunol. Res..

[128] Briere D., Sudhakar N., Woods D.M., Hallin J., Engstrom L.D., Aranda R., Chiang H., Sodre A.L., Olson P., Weber J.S. (2018). The class I/IV HDAC inhibitor mocetinostat increases tumor antigen presentation, decreases immune suppressive cell types and augments checkpoint inhibitor therapy. Cancer Immunol. Immunother..

[129] Kim Y.-D., Park S.-M., Ha H.C., Lee A.R., Won H., Cha H., Cho S., Cho J.M. (2020). HDAC Inhibitor, CG-745, Enhances the Anti-Cancer Effect of Anti-PD-1 Immune Checkpoint Inhibitor by Modulation of the Immune Microenvironment. J. Cancer.

[130] Vogelmann A., Robaa D., Sippl W., Jung M. (2020). Proteolysis targeting chimeras (PROTACs) for epigenetics research. Curr. Opin. Chem. Biol..

[131] Liu J.-R., Yu C.-W., Hung P.-Y., Hsin L.-W., Chern J.-W. (2019). High-selective HDAC6 inhibitor promotes HDAC6 degradation following autophagy modulation and enhanced antitumor immunity in glioblastoma. Biochem. Pharmacol..

[132] Cao Z., Gu Z., Lin S., Chen D., Wang J., Zhao Y., Li Y., Liu T., Li Y., Wang Y. (2021). Attenuation of NLRP3 Inflammasome Activation by Indirubin-Derived PROTAC Targeting HDAC6. ACS Chem. Biol..

[133] Lienlaf M., Perez-Villarroel P., Knox T., Pabon M., Sahakian E., Powers J., Woan K.V., Lee C., Cheng F., Deng S. (2016). Essential role of HDAC6 in the regulation of PD-L1 in melanoma. Mol. Oncol..

[134] Keremu A., Aimaiti A., Liang Z., Zou X. (2019). Role of the HDAC6/STAT3 pathway in regulating PD-L1 expression in osteosarcoma cell lines. Cancer Chemother. Pharmacol..

[135] Yang K., Zhao Y., Nie X., Wu H., Wang B., Almodovar-Rivera C.M., Xie H., Tang W. (2020). A Cell-Based Target Engagement Assay for the Identification of Cereblon E3 Ubiquitin Ligase Ligands and Their Application in HDAC6 Degraders. Cell Chem. Biol..

[136] Ahmed A., Tait S.W.G. (2020). Targeting immunogenic cell death in cancer. Mol. Oncol..

[137] Chong S.J.F., Marchi S., Petroni G., Kroemer G., Galluzzi L., Pervaiz S. (2020). Noncanonical Cell Fate Regulation by Bcl-2 Proteins. Trends Cell Biol..

[138] Garcia-Aranda M., Perez-Ruiz E., Redondo M. (2018). Bcl-2 Inhibition to Overcome Resistance to Chemo- and Immunotherapy. Int. J. Mol. Sci..

[139] Ludwig L.M., Nassin M.L., Hadji A., LaBelle J.L. (2016). Killing Two Cells with One Stone: Pharmacologic BCL-2 Family Targeting for Cancer Cell Death and Immune Modulation. Front. Pediatr..

[140] Begley J., Vo D.D., Morris L.F., Bruhn K.W., Prins R.M., Mok S., Koya R.C., Garban H.J., Comin-Anduix B., Craft N. (2009). Immunosensitization with a Bcl-2 small molecule inhibitor. Cancer Immunol. Immunother..

[141] Lickliter J.D., Cox J., McCarron J., Martinez N.R., Schmidt C.W., Lin H., Nieda M., Nicol A.J. (2007). Small-molecule Bcl-2 inhibitors sensitise tumour cells to immune-mediated destruction. Br. J. Cancer.

[142] Kim P.S., Jochems C., Grenga I., Donahue R.N., Tsang K.Y., Gulley J.L., Schlom J., Farsaci B. (2014). Pan-Bcl-2 inhibitor, GX15-070 (obatoclax), decreases human T regulatory lymphocytes while preserving effector T lymphocytes: A rationale for its use in combination immunotherapy. J. Immunol..

[143] Farsaci B., Sabzevari H., Higgins J.P., di Bari M.G., Takai S., Schlom J., Hodge J.W. (2010). Effect of a small molecule BCL-2 inhibitor on immune function and use with a recombinant vaccine. Int. J. Cancer.

[144] Kim P.S., Schlom J. (2014). Potential utility of the pan-Bcl-2 inhibitor GX15-070 (obatoclax) in cancer immunotherapy. OncoImmunology.

[145] Khan S., Wiegand J., Zhang P., Hu W., Thummuri D., Budamagunta V., Hua N., Jin L., Allegra C.J., Kopetz S.E. (2022). BCL-XL PROTAC degrader DT2216 synergizes with sotorasib in preclinical models of KRAS(G12C)-mutated cancers. J. Hematol. Oncol..

[146] Qi S.M., Dong J., Xu Z.Y., Cheng X.D., Zhang W.D., Qin J.J. (2021). PROTAC: An Effective Targeted Protein Degradation Strategy for Cancer Therapy. Front. Pharmacol..

[147] Zhang Q., Khan S., Zhang X., Kuruvilla V.M., Ghotbaldini S., Wells J., Baran N., Cai T., Han L., Ferrando A. (2019). Targeting BCL-XL By Protac DT2216 Effectively Eliminates Leukemia Cells in T-ALL Pre-Clinical Models. Blood.

[148] He Y., Koch R., Budamagunta V., Zhang P., Zhang X., Khan S., Thummuri D., Ortiz Y.T., Zhang X., Lv D. (2020). DT2216-a Bcl-xL-specific degrader is highly active against Bcl-xL-dependent T cell lymphomas. J. Hematol. Oncol..

[149] Jia Y., Zhang Q., Zhang W., Andreeff M., Jain N., Zhang P., Zheng G., Zhou D., Konopleva M. (2021). Targeting BCL-XL and BCL-2 By Protac 753B Effectively Eliminates AML Cells and Enhances Efficacy of Chemotherapy by Targeting Senescent Cells. Blood.

[150] Li M., Wang D., He J., Chen L., Li H. (2020). Bcl-XL: A multifunctional anti-apoptotic protein. Pharmacol. Res..

[151] Dong J., Cheng X.D., Zhang W.D., Qin J.J. (2021). Recent Update on Development of Small-Molecule STAT3 Inhibitors for Cancer Therapy: From Phosphorylation Inhibition to Protein Degradation. J. Med. Chem..

[152] Qin J.J., Yan L., Zhang J., Zhang W.D. (2019). STAT3 as a potential therapeutic target in triple negative breast cancer: A systematic review. J. Exp. Clin. Cancer Res..

[153] Qin J., Shen X., Zhang J., Jia D. (2020). Allosteric inhibitors of the STAT3 signaling pathway. Eur. J. Med. Chem..

[154] Mohrherr J., Uras I.Z., Moll H.P., Casanova E. (2020). STAT3: Versatile Functions in Non-Small Cell Lung Cancer. Cancers.

[155] Zou S., Tong Q., Liu B., Huang W., Tian Y., Fu X. (2020). Targeting STAT3 in Cancer Immunotherapy. Mol. Cancer.

[156] Wang T., Niu G., Kortylewski M., Burdelya L., Shain K., Zhang S., Bhattacharya R., Gabrilovich D., Heller R., Coppola D. (2004). Regulation of the innate and adaptive immune responses by Stat-3 signaling in tumor cells. Nat. Med..

[157] Huang Q., Zhong Y., Dong H., Zheng Q., Shi S., Zhu K., Qu X., Hu W., Zhang X., Wang Y. (2020). Revisiting signal transducer and activator of transcription 3 (STAT3) as an anticancer target and its inhibitor discovery: Where are we and where should we go?. Eur. J. Med. Chem..

[158] Khan M.W., Saadalla A., Ewida A.H., Al-Katranji K., Al-Saoudi G., Giaccone Z.T., Gounari F., Zhang M., Frank D.A., Khazaie K. (2018). The STAT3 inhibitor pyrimethamine displays anti-cancer and immune stimulatory effects in murine models of breast cancer. Cancer Immunol. Immunother..

[159] Akiyama Y., Nonomura C., Ashizawa T., Iizuka A., Kondou R., Miyata H., Sugino T., Mitsuya K., Hayashi N., Nakasu Y. (2017). The anti-tumor activity of the STAT3 inhibitor STX-0119 occurs via promotion of tumor-infiltrating lymphocyte accumulation in temozolomide-resistant glioblastoma cell line. Immunol. Lett..

[160] Pei J., Zhang Y., Luo Q., Zheng W., Li W., Zeng X., Li Q., Quan J. (2019). STAT3 inhibition enhances CDN-induced STING signaling and antitumor immunity. Cancer Lett..

[161] Proia T.A., Singh M., Woessner R., Carnevalli L., Bommakanti G., Magiera L., Srinivasan S., Grosskurth S., Collins M., Womack C. (2020). STAT3 Antisense Oligonucleotide Remodels the Suppressive Tumor Microenvironment to Enhance Immune Activation in Combination with Anti-PD-L1. Clin. Cancer Res..

[162] Kumar S., Principe D.R., Singh S.K., Viswakarma N., Sondarva G., Rana B., Rana A. (2020). Mitogen-Activated Protein Kinase Inhibitors and T-Cell-Dependent Immunotherapy in Cancer. Pharmaceuticals.

[163] Kuhnol C., Herbarth M., Foll J., Staege M.S., Kramm C. (2013). CD137 stimulation and p38 MAPK inhibition improve reactivity in an in vitro model of glioblastoma immunotherapy. Cancer Immunol. Immunother..

[164] Stutvoet T.S., Kol A., de Vries E.G., de Bruyn M., Fehrmann R.S., van Scheltinga A.G.T., de Jong S. (2019). MAPK pathway activity plays a key role in PD-L1 expression of lung adenocarcinoma cells. J. Pathol..

[165] Lu Y., Zhang M., Wang S., Hong B., Wang Z., Li H., Zheng Y., Yang J., Davis R.E., Qian J. (2014). p38 MAPK-inhibited dendritic cells induce superior antitumour immune responses and overcome regulatory T-cell-mediated immunosuppression. Nat. Commun..

[166] Dushyanthen S., Teo Z.L., Caramia F., Savas P., Mintoff C.P., Virassamy B., Henderson M.A., Luen S.J., Mansour M., Kershaw M.H. (2017). Agonist immunotherapy restores T cell function following MEK inhibition improving efficacy in breast cancer. Nat. Commun..

[167] Mehrotra S., Chhabra A., Chattopadhyay S., Dorsky D.I., Chakraborty N.G., Mukherji B. (2004). Rescuing melanoma epitope-specific cytolytic T lymphocytes from activation-induced cell death, by SP600125, an inhibitor of JNK: Implications in cancer immunotherapy. J. Immunol..

[168] Ebert P.J.R., Cheung J., Yang Y., McNamara E., Hong R., Moskalenko M., Gould S.E., Maecker H., Irving B.A., Kim J.M. (2016). MAP Kinase Inhibition Promotes T Cell and Anti-tumor Activity in Combination with PD-L1 Checkpoint Blockade. Immunity.

[169] Wang C., Wang H., Zheng C., Liu Z., Gao X., Xu F., Niu Y., Zhang L., Xu P. (2021). Research progress of MEK1/2 inhibitors and degraders in the treatment of cancer. Eur. J. Med. Chem..

[170] Yu W.B., Ye Z.H., Chen X., Shi J.J., Lu J.J. (2021). The development of small-molecule inhibitors targeting CD47, Drug Discov Today. Drug Discov. Today.

[171] Borsari C., Trader D.J., Tait A., Costi M.P. (2020). Designing Chimeric Molecules for Drug Discovery by Leveraging Chemical Biology. J. Med. Chem..

[172] Ye P., Chi X., Cha J.H., Luo S., Yang G., Yan X., Yang W.H. (2021). Potential of E3 Ubiquitin Ligases in Cancer Immunity: Opportunities and Challenges. Cells.

[173] Huang J., Wang S., Jia Y., Zhang Y., Dai X., Li B. (2020). Targeting FOXP3 complex ensemble in drug discovery. Adv. Protein Chem. Struct. Biol..

[174] Zhang W., Liu X., Zhu Y., Liu X., Gu Y., Dai X., Li B. (2021). Transcriptional and posttranslational regulation of Th17/Treg balance in health and disease. Eur. J. Immunol..

[175] Li J., Zhang P., Zhou M., Liu C., Huang Y., Li L. (2022). Trauma-Responsive Scaffold Synchronizing Oncolysis Immunization and Inflammation Alleviation for Post-Operative Suppression of Cancer Metastasis. ACS Nano.

[176] Yi X., Yan Y., Li L., Zhou R., Shen X., Huang Y. (2022). Combination of mitochondria impairment and inflammation blockade to combat metastasis. J. Control. Release.

[177] Tang F., Tie Y., Hong W., Wei Y., Tu C., Wei X. (2021). Targeting Myeloid-Derived Suppressor Cells for Premetastatic Niche Disruption After Tumor Resection. Ann. Surg. Oncol..

[178] Hong W., Mo F., Zhang Z., Huang M., Wei X. (2020). Nicotinamide Mononucleotide: A Promising Molecule for Therapy of Diverse Diseases by Targeting NAD+ Metabolism. Front. Cell Dev. Biol..

[179] Zhou M., Zuo Q., Huang Y., Li L. (2022). Immunogenic hydrogel toolkit disturbing residual tumor “seeds” and pre-metastatic “soil” for inhibition of postoperative tumor recurrence and metastasis. Acta Pharm. Sin. B.

[180] Chen R., Jing J., Siwko S., Huang Y., Zhou Y. (2020). Intelligent cell-based therapies for cancer and autoimmune disorders. Curr. Opin. Biotechnol..

[181] Lee S.M., Kang C.H., Choi S.U., Kim Y., Hwang J.Y., Jeong H.G., Park C.H. (2020). A Chemical Switch System to Modulate Chimeric Antigen Receptor T Cell Activity through Proteolysis-Targeting Chimaera Technology. ACS Synth. Biol..

[182] Huang R., Jing X., Huang X., Pan Y., Fang Y., Liang G., Liao Z., Wang H., Chen Z., Zhang Y. (2020). Bifunctional Naphthoquinone Aromatic Amide-Oxime Derivatives Exert Combined Immunotherapeutic and Antitumor Effects through Simultaneous Targeting of Indoleamine-2,3-dioxygenase and Signal Transducer and Activator of Transcription 3. J. Med. Chem..

[183] Zheng M., Huo J., Gu X., Wang Y., Wu C., Zhang Q., Wang W., Liu Y., Liu Y., Zhou X. (2021). Rational Design and Synthesis of Novel Dual PROTACs for Simultaneous Degradation of EGFR and PARP. J. Med. Chem..

[184] Girardini M., Maniaci C., Hughes S.J., Testa A., Ciulli A. (2019). Cereblon versus VHL: Hijacking E3 ligases against each other using PROTACs. Bioorg. Med. Chem..

[185] Maniaci C., Hughes S.J., Testa A., Chen W., Lamont D.J., Rocha S., Alessi D.R., Romeo R., Ciulli A. (2017). Homo-PROTACs: Bivalent small-molecule dimerizers of the VHL E3 ubiquitin ligase to induce self-degradation. Nat. Commun..

[186] Kuwahara-Ota S., Shimura Y., Steinebach C., Isa R., Yamaguchi J., Nishiyama D., Fujibayashi Y., Takimoto-Shimomura T., Mizuno Y., Matsumura-Kimoto Y. (2020). Lenalidomide and pomalidomide potently interfere with induction of myeloid-derived suppressor cells in multiple myeloma. Br. J. Haematol..

[187] Zhao L., Zhao J., Zhong K., Tong A., Jia D. (2022). Targeted protein degradation: Mechanisms, strategies and application. Signal Transduct. Target. Ther..

[188] Zhong Y., Chi F., Wu H., Liu Y., Xie Z., Huang W., Shi W., Qian H. (2022). Emerging targeted protein degradation tools for innovative drug discovery: From classical PROTACs to the novel and beyond. Eur. J. Med. Chem..

[189] Chen J., Qiu M., Ma F., Yang L., Glass Z., Xu Q. (2021). Enhanced protein degradation by intracellular delivery of pre-fused PROTACs using lipid-like nanoparticles. J. Control. Release.

[190] Chen Y., Tandon I., Heelan W., Wang Y., Tang W., Hu Q. (2022). Proteolysis-targeting chimera (PROTAC) delivery system: Advancing protein degraders towards clinical translation. Chem. Soc. Rev..

